# Plastid NDH Pseudogenization and Gene Loss in a Recently Derived Lineage from the Largest Hemiparasitic Plant Genus *Pedicularis* (Orobanchaceae)

**DOI:** 10.1093/pcp/pcab074

**Published:** 2021-05-28

**Authors:** Xin Li, Jun-Bo Yang, Hong Wang, Yu Song, Richard T Corlett, Xin Yao, De-Zhu Li, Wen-Bin Yu

**Affiliations:** Center for Integrative Conservation, Xishuangbanna Tropical Botanical Garden, Chinese Academy of Sciences, Mengla, Yunnan 666303, China; Center of Conservation Biology, Core Botanical Gardens, Chinese Academy of Sciences, Mengla, Yunnan 666303, China; University of Chinese Academy of Sciences, Shijingshan District, Beijing 100049, China; Plant Germplasm and Genomics Center, Germplasm Bank of Wild Species, Kunming Institute of Botany, Chinese Academy of Sciences, Kunming, Yunnan 650201, China; Key Laboratory for Plant Diversity and Biogeography of East Asia, Kunming Institute of Botany, Chinese Academy of Sciences, Kunming, Yunnan 650201, China; Center for Integrative Conservation, Xishuangbanna Tropical Botanical Garden, Chinese Academy of Sciences, Mengla, Yunnan 666303, China; Center of Conservation Biology, Core Botanical Gardens, Chinese Academy of Sciences, Mengla, Yunnan 666303, China; Southeast Asia Biodiversity Research Institute, Chinese Academy of Sciences, Yezin, Nay Pyi Taw 05282, Myanmar; Center for Integrative Conservation, Xishuangbanna Tropical Botanical Garden, Chinese Academy of Sciences, Mengla, Yunnan 666303, China; Center of Conservation Biology, Core Botanical Gardens, Chinese Academy of Sciences, Mengla, Yunnan 666303, China; Center for Integrative Conservation, Xishuangbanna Tropical Botanical Garden, Chinese Academy of Sciences, Mengla, Yunnan 666303, China; Center of Conservation Biology, Core Botanical Gardens, Chinese Academy of Sciences, Mengla, Yunnan 666303, China; Plant Germplasm and Genomics Center, Germplasm Bank of Wild Species, Kunming Institute of Botany, Chinese Academy of Sciences, Kunming, Yunnan 650201, China; Center for Integrative Conservation, Xishuangbanna Tropical Botanical Garden, Chinese Academy of Sciences, Mengla, Yunnan 666303, China; Center of Conservation Biology, Core Botanical Gardens, Chinese Academy of Sciences, Mengla, Yunnan 666303, China; Southeast Asia Biodiversity Research Institute, Chinese Academy of Sciences, Yezin, Nay Pyi Taw 05282, Myanmar

**Keywords:** Hemiparasite, IR expansion, NA(D)H dehydrogenase-like complex, Orobanchaceae, Pseudogenization, Relaxation of selection

## Abstract

The plastid genome (plastome) is highly conserved in both gene order and content and has a lower mutation rate than the nuclear genome. However, the plastome is more variable in heterotrophic plants. To date, most such studies have investigated just a few species or only holoheterotrophic groups, and few have examined plastome evolution in recently derived lineages at an early stage of transition from autotrophy to heterotrophy. In this study, we investigated the evolutionary dynamics of plastomes in the monophyletic and recently derived *Pedicularis* sect. *Cyathophora* (Orobanchaceae). We obtained 22 new plastomes, 13 from the six recognized species of section *Cyathophora*, six from hemiparasitic relatives and three from autotrophic relatives. Comparative analyses of gene content, plastome structure and selection pressure showed dramatic differences among species in section *Cyathophora* and in *Pedicularis* as a whole. In comparison with autotrophic relatives and other *Pedicularis* spp., we found that the inverted repeat (IR) region in section *Cyathophora* had expansions to the small single-copy region, with a large expansion event and two independent contraction events. Moreover, NA(D)H dehydrogenase, *accD* and *ccsA* have lost function multiple times, with the function of *accD* being replaced by nuclear copies of an *accD*-like gene in *Pedicularis* spp. The *ccsA* and *ndhG* genes may have evolved under selection in association with IR expansion/contraction events. This study is the first to report high plastome variation in a recently derived lineage of hemiparasitic plants and therefore provides evidence for plastome evolution in the transition from autotrophy to heterotrophy.

## Introduction

Plastids are key organelles for photosynthesis in green plants. The plastid possesses its own genome in a single circular molecule. The plastid genome (or plastome) is 140–180 kb in size and has a quadripartite structure, including two inverted repeat (IR) regions separated by a large single-copy (LSC) region and a small single-copy (SSC) region (Palmer 1983, Palmer et al. 1987, Wicke et al. 2011, Ruhlman and Jansen 2014). The IR regions are highly conserved in size and gene content among angiosperms, but IR expansions and contractions (and even complete losses) have been reported in several distant lineages (Zhu et al. 2016, Mower and Vickrey 2018). Some studies have demonstrated that genes in the IR region have lower substitution rates than genes in the single-copy regions, which may help to stabilize the plastome structure (Wicke et al. 2011, Zhu et al. 2016). Generally, the plastome contains about 114 unique genes, including 80 coding sequence (CDS) genes, 30 transfer RNA (tRNA) genes and four ribosomal RNA (rRNA) genes (Wicke et al. 2011). However, heterotrophic plants, including haustorial parasites and mycoheterotrophs, no longer need to photosynthesize and may have degraded plastomes resulting from gene loss and structural changes (Graham et al. 2017, Wicke and Naumann 2018).

From a comprehensive survey of land plants, Wicke and Naumann (2018) have documented that heterotrophic plants occur in at least 23 orders. According to their carbon acquisition pathways, heterotrophic plants can be classified into mycoheterotrophs, which acquire carbon from associated mycorrhizal fungi (Leake 1994), and parasites, which acquire carbon from other land plants using a specialized haustorium (Yoshida et al. 2016). To date, parasitic plants have been found in 12 orders of angiosperms. Around 95% of species belong to the Orobanchaceae (Lamiales) and Santalales, and the remaining 5% belong to Apodanthaceae (Cucurbitales), *Cassytha* L. (Laurales), *Cuscuta* L. (Solanales), Cynomoriaceae (Saxifragales), Cytinaceae (Malvales), Hydnoraceae (Piperales), Krameriaceae (Zygophyllales), Lennoaceae (Boraginales), Mitrastemonaceae (Ericales) and Rafflesiaceae (Malpighiales) (Yoshida et al. 2016). Plant parasitism is characterized by a transition from autotrophy to partial (hemiparasite) or full parasitism (holoparasite). The plastomes of parasitic plants degrade as they transition from hemiparasitism to holoparasitism; this transition is accompanied by pseudogenization or by complete loss of photosynthesis-related genes (Wicke and Naumann 2018). It has been demonstrated that plastid genes experienced relaxed functional constraints during the transition from autotrophy to heterotrophy Orobanchaceae (Wolfe et al. 1992, Wicke et al. 2016), but the impact of the transition from autotrophy to hemiparasitism has not been well documented.

Plastome degradation in parasitic plants may include structural reconfiguration, IR expansion or contraction, or complete loss. For example, plastomes of *Pedicularis ishidoyana* Koidz. & Ohwi, *Buchnera americana* L., *Schwalbea americana* L. and *Striga* spp. (*S. aspera* Benth., *S. forbessii* Benth. and *S. hermonthica* Benth.) have experienced IR expansion to the LSC and/or SSC regions (Wicke et al. 2013, Cho et al. 2018, Frailey et al. 2018); *Cassytha* has lost one IR region (Song et al. 2017) and some Santalales species have a large inversion in the LSC region (Chen et al. 2019). Published plastome data from distantly related lineages of hemiparasitic plants show that some plastid NA(D)H dehydrogenase-like (NDH) genes have been independently lost or reside as nonfunctional pseudogenes (see **Supplementary Table S1**). For example, plastomes of *Cassytha* spp. (Laurales) have five NDH pseudogenes (*ndhB, ndhD, ndhE, ndhF* and *ndhH*) and have completely lost the other six NDH genes (Song et al. 2017, Wu et al. 2017). Similarly, plastomes of many hemiparasitic Orobanchaceae have *ndhB, ndhD, ndhE, ndhF* and *ndhH* pseudogenes (Wicke et al. 2016, Frailey et al. 2018), and plastomes of hemiparasitic Santalales have completely lost *ndhA, ndhC, ndhG, ndhI, ndhJ* and *ndhK* (Petersen et al. 2015, Chen et al. 2019). It is therefore not possible to trace the evolutionary history of functional or physical losses of plastid NDH genes by comparative analyses of distantly related hemiparasitic lineages. In contrast, gradual pseudogenization, fragmentation and loss of plastid NDH genes can be observed by comparative analyses of plastomes from the same genus or a recently derived lineage.

*Pedicularis* L. is the largest hemiparasitic genus of Orobanchaceae (including approximately 700 species) (Li 1951, Fischer 2004) and is well-known for its floral variation. It is widely distributed in the north temperate zone with more than two-thirds of the species represented in the Hengduan Mountains of south-central China (Yang et al. 1998). The roots form haustoria through xylem connections with host roots (Ren et al. 2010, Li et al. 2012). Recent molecular phylogenetic studies show that the genus *Pedicularis* is monophyletic and includes 13 major clades (Ree 2005, Tkach et al. 2014, Yu et al. 2015). *Pedicularis* sect. *Cyathophora*Li (1948) is a monophyletic group in clade IV, fully supported by both morphological characters and molecular phylogenies, which diverged at around 7.14 Mya (Eaton and Ree 2013, Yu et al. 2013, Wang et al. 2017). This recently derived lineage is characterized by having cup-like bracts around the stem at each node, and includes all corolla types found in *Pedicularis*, making it an ideal group for investigation of species diversification and plastome evolution in the genus (Eaton and Ree 2013, Yu et al. 2013).

In this study, we sampled plastomes from all six recognized species in *Pedicularis* sect. *Cyathophora* [*P. connata* H.L.Li, *P. cyathophylla* Franch., *P. cyathophylloides* Limpr., *P. rex* C.B.Clarke ex Maxim., *P. superba* Franch. ex Maxim. and *P. thamnophila* (Hand.-Mazz.) H.L.Li]. We also sampled plastomes of five species from other sections of *Pedicularis* (*P. przewalskii* Maxim., *P. insignis* Bonati, *P. lyrata* Prain, *P. tongolensis* Franch., *P. kangdinggensis* Tsoong), the closely related hemiparasite, *Phtheirospermum japonicum* (Thunb.) Kanitz, in tribe Pedicularideae (Orobanchaceae), as well as three autotrophic relatives, *Lindenbergia muraria* (Roxburgh ex D. Don) Bruhl (Orobanchaceae), *Rehmannia glutinosa* (Gaertn.) Steud. (Orobanchaceae) and *Paulownia tomentosa* (Thunb.) Steud. (Paulowniaceae). The main objectives of this study were (i) to reconstruct the phylogenetic relationships of *Pedicularis* sect. *Cyathophora* using the complete plastome sequences; (ii) to investigate plastome variation in section *Cyathophora* and (iii) to trace evolution of NDH, *acc*D and *ccs*A.

## Results

### Phylogenomic analysis

**Fig. 1:**
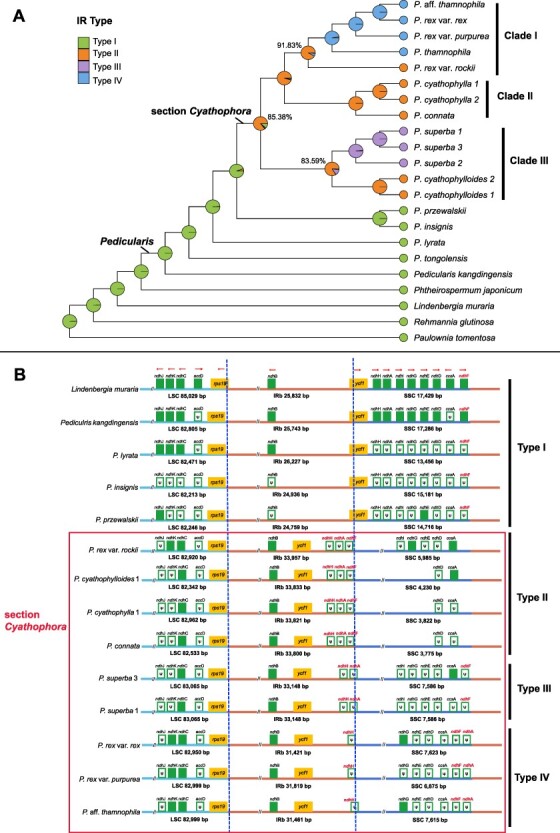
Phylogenomic relationship and evolutionary history of the five types of IR structure in *Pedicularis* sect. *Cyathophora* and other relatives (A). Variations of the IR region boundary and the distribution of *accD, ccsA* and NDH genes (B). Pseudogenes are marked by ψ in the open boxes, and direction of transcription is indicated by an arrow. The key ‘landmark’ genes, *ndhA, ndhF* and *ndhH*, are annotated in red font.

The monophyly of section *Cyathophora* was strongly supported (BS (Bootstrap support value) = 100; **Fig. 1**, **Supplementary Fig. S1**). The species of *Cyathophora* were divided into three main clades: clade I corresponds to series *Reges* (BS = 100), including the three infraspecific taxa of *P. rex, P. thamnophila*, and an unknown taxon, in which var. *rockii* is sister to the other four taxa; clade II contains *P. cyathophylla* and *P. connata* (BS = 100); clade III contains *P. cyathophylloides* and *P. superba* (BS = 100).

### Characteristics of the plastid genomes

The plastomes of 18 *Pedicularis* and four non-*Pedicularis* species displayed the typical quadripartite structure. Plastomes of *Pedicularis* species varied from 146,480 to 157,028 bp. The lengths of the four regions varied as follows: LSC 82,213–83,342 bp; SSC 3,738 to 17,286 bp; IR 24,759–33,810 bp (**Table 1**). The plastome sequences of four non-*Pedicularis* species varied considerably less, from 153,372 to 154,798 bp: LSC 84,478–85,438 bp; SSC 17,429–17,732 bp; IR 25,608–25,832 bp (**Table 1**).

**Table 1:** Overview of physical properties of plastid chromosomes in non-parasitic and parasitic plants (*Phtheirospermum* and *Pedicularis*)

	Genome size	LSC	IR	SSC	Gene content	
Species	(bp GC%)	(bp GC%)	(bp GC%)*a*	(bp GC%)	(CDS*a*/tRNA*a*/rRNA*a*)	Pseudogenes/loss*b*
*Pedicularis* sect. *Cyathophora*						
Series *Cyathophyllae*						
*P. cyathophylla* 1	154,426 (38.3)	82,962 (36.6)	33,821 (40.7)	3,822 (33.9)	69/4/30	*ndh*A^2^,D^2^,E^3^,F^2^,G^3^,H^1^,I^3^,J^1^,K^1^; *acc*D^1^; *ccs*A^1^
*P. cyathophylla* 2	154,412 (38.3)	82,972 (36.6)	33,861 (40.7)	3,718 (33.6)	69/4/30	*ndh*A^2^,D^2^,E^3^,F^2^,G^3^,H^1^,I^3^,J^1^,K^1^; *acc*D^1^; *ccs*A^1^
Series *Cyathophylloides*						
*P. cyathophylloides* 1	154,238 (38.3)	82,342 (36.6)	33,833 (40.7)	4,230 (34.3)	70/4/30	*ndh*A^2^,D^1^,E^3^,F^2^,G^3^,H^1^,I^3^,J^2^,K^2^; *acc*D^1^
*P. cyathophylloides* 2	154,238 (38.3)	83,342 (36.6)	33,833 (40.7)	4,230 (34.3)	70/4/30	*ndh*A^2^,D^1^,E^3^,F^2^,G^3^,H^1^,I^3^,J^1^,K^1^; *acc*D^2^
Series *Reges*						
*P. rex* var. *rex*	153,415 (38.3)	82,950 (36.6)	31,421 (41.1)	7,623 (34.2)	70/4/30	*ndh*A^2^,D^1^,E^1^,F^2^,G^2^,H^1^,I^3^,J^1^; *acc*D^1^; *ccs*A^1^
*P. rex var. purpurea*	153,512 (38.3)	82,999 (36.6)	31,819 (41.1)	6,875 (34.0)	70/4/30	*ndh*A^2^,D^1^,E^1^,F^2^,G^2^,H^1^,I^3^,J^1^; *acc*D^1^; *ccs*A^1^
*P. rex* var. *rockii*	156,819 (38.2)	82,920 (36.6)	33,957 (40.6)	5,985 (33.8)	71/4/30	*ndh*A^2^,D^1^,E^1^,F^2^, H^1^,I^1^,J^1^; *acc*D^1^; *ccs*A^1^
*P.* aff. *thamnophila*	153,446 (38.3)	82,999 (36.6)	31,416 (41.1)	7,615 (34.2)	71/4/30	*ndh*A^3^,D^1^,E^1^,F^2^,H^2^,I^3^,J^1^; *acc*D^1^; *ccs*A^1^
*P. thamnophila*	153,612 (38.3)	82,972 (36.6)	31,411 (41.1)	7,818 (34.3)	71/4/30	*ndh*A^3^,D^1^,E^1^,F^2^,H^2^,I^3^,J^1^; *acc*D^1^; *ccs*A^1^
Series *Superbae*						
*P. connata*	153,908 (38.4)	82,533 (36.6)	33,800 (40.7)	3,775 (33.9)	69/4/30	*ndh*A^2^,D^2^,E^3^,F^2^,G^3^,H^1^,I^3^,J^1^,K^1^; *acc*D^1^; *ccs*A^1^
*P. superba* 1	157,028 (38.2)	83,056 (36.6)	33,148 (40.9)	7,676 (33.2)	69/4/30	*ndh*A^1^,D^1^,E^1^,F^2^,G^1^,H^1^,I^1^,J^1^,K^1^; *acc*D^1^; *ccs*A^1^
*P. superba* 2	156,942 (38.2)	83,061 (36.6)	33,079 (40.9)	7,723 (33.2)	70/4/30	*ndh*A^1^,D^1^,E^1^,F^2^,G^1^,H^1^,I^1^,J^1^, K^1^; *acc*D^1^
*P. superba* 3	156,947 (38.2)	83,065 (36.6)	33,148 (40.9)	7,586 (33.4)	70/4/30	*ndh*A^1^,D^1^,E^1^,F^2^,G^1^,H^1^,I^1^,J^1^, K^1^; *acc*D^1^
Others *Pedicularis*						
*P. przewalskii*	146,480 (38.5)	82,246 (36.6)	24,759 (43.5)	14,716 (32.8)	69/4/30	*ndh*A^2^,B^1^,C^2^,D^1^,F^3^,G^2^,H^1^,I^1^,K^2^; *acc*D^1^; *ccs*A^1^
*P. insignis*	147,267 (38.6)	82,213 (36.7)	24,936 (43.5)	15,181 (32.7)	67/4/30	*ndh*A^2^,B^1^,C^1^,D^2^,E^1^,F^2^,G^1^,H^1^,I^1^,J^1^,K^1^; *acc*D^2^; *ccs*A^2^
*P. lyrata*	148,379 (38.4)	82,471 (36.6)	26,227 (43.0)	13,456 (31.9)	71/4/30	*ndh*A^1^,D^2^,E^1^,F^2^,G^1^,H^1^,I^1^; *acc*D^2^; *ccs*A^1^
*P. tongolensis*	151,850 (38.3)	83,240 (36.4)	25,729 (43.3)	17,251 (32.5)	79/4/30	*ccs*A^1^
*P. kangdingensis*	151,577 (38.3)	82,805 (36.4)	25,743 (43.3)	17,286 (32.4)	79/4/30	*ccs*A^1^
Outgroup						
*Phtheirospermum japonicum*	153,372 (38.3)	84,478 (36.4)	25,608 (43.4)	17,678 (32.3)	80/4/30	
*Lindenbergia muraria*	154,122 (37.7)	85,029 (35.7)	25,832 (43.2)	17,429 (31.5)	80/4/30	
*Paulownia tomentosa*	154,798 (38.0)	85,438 (36.0)	25,814 (43.2)	17,732 (32.4)	80/4/30	
*Rehmannia glutinosa*	153,777 (37.9)	84,669 (36.0)	25,759 (43.1)	17,590 (32.2)	80/4/30	

^a^ Number of unique genes.

^b^ States of pseudogenes or complete loss: 1, full sequence with premature stop codon; 2, truncated gene; 3, complete loss.

The gene content of the plastome varied from 111 to 114 unique genes in *Pedicularis*, including 67–80 CDS, 30 tRNA, four rRNA genes, and one to 13 pseudogenes (**Table 1**, **Supplementary Fig. S2**). The non-*Pedicularis* plastomes contained 114 unique genes, including 80 CDS genes, 30 tRNA genes and four rRNA genes (**Table 1**, **Supplementary Fig. S2**). Of these, nine CDS genes, four rRNA genes and seven tRNA genes were duplicated in the IR regions.

**Fig. 2:**
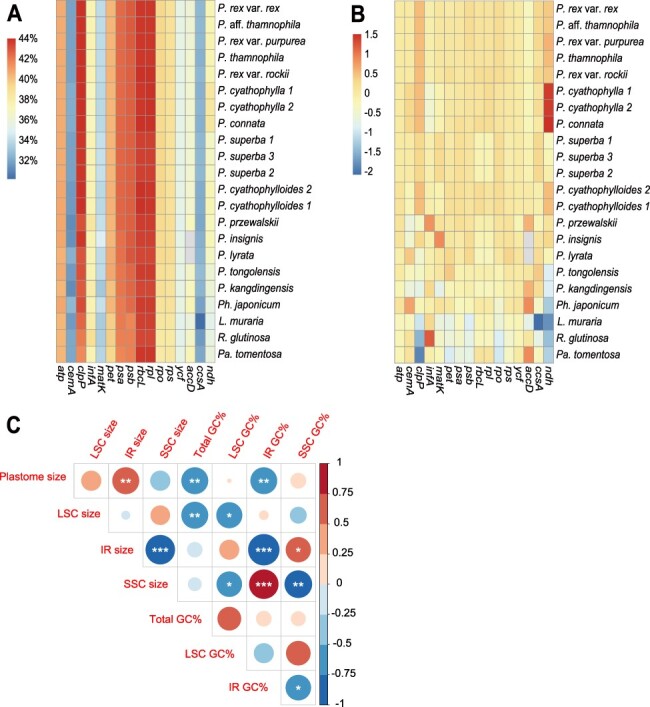
Heatmap of the GC content by gene groups or genes in all species (A), the deviation values from the average GC content of each gene group or gene (B) and correlation analysis among the GC content and plastome/region size (C). Significance levels: ***P < 0.001; **P < 0.01; *P < 0.05.

The GC contents of 18 *Pedicularis* plastomes ranged from 38.2 to 38.6%, with the LSC, SSC and IR regions at 36.4–36.7%, 40.6–43.5% and 31.9–34.3%, respectively (**Table 1**). The GC contents of non-*Pedicularis* plastomes were 37.7 to 38.3%, with the LSC, SSC and IR regions at 35.7–36.4%, 31.5–32.4% and 43.1–43.4%, respectively (**Table 1**). The GC content was above 40% in *atp, clp, pet, psa, psb, rpl* and *rbcL* in all platomes (**Fig. 2A**). The GC content of NDH genes was higher than average in section *Cyathophora* (**Fig. 2B**). Plastome size was negatively correlated with GC content of both the whole plastome (*r* = −0.72, *P *< 0.01) and the IR region (*r* = −0.72, *P *< 0.01) and was positively correlated with IR size (*r* = 0.73, *P *< 0.01) (**Fig. 2C**). LSC region size was negatively correlated with plastome GC content (*r* = −0.72, *P* < 0.01) (**Fig. 2C**). IR region size was negatively correlated with the SSC region size (*r* = −0.87, *P* < 0.001) and its own GC content (*r* = −0.99, *P* < 0.001) (**Fig. 2C**), because the IR region has captured adjacent SSC genes in *Pedicularis* spp. The size of the SSC region was negative correlated with its own GC content (*r* = −0.76, *P* < 0.001) and positively correlated with the GC content of the IR region (*r* = 0.87, *P* < 0.01) (**Fig. 2C**). Section *Cyathophora* plastomes were smaller than other *Pedicularis* species (*W* = 65.0, *P* < 0.01), and *Pedicularis* plastomes were smaller than those of other genera (*W* =20.0, *P* < 0.05; **Supplementary Table S3**). Section *Cyathophora* IR and SSC regions were, respectively, larger and smaller than those of both other *Pedicularis* (IR size: *W *= 65.0, *P *< 0.01; SSC size: *W *= 0.0, *P *< 0.01) and non-*Pedicularis* (IR size: *W *= 52.0, *P *< 0.01; SSC size: *W *= 0.0, *P *< 0.01). The same trend held for IR and SSC GC content. Section *Cyathophora* also had fewer genes and more pseudogenes than other *Pedicularis* species (CDS genes: *W *= 20.0, *P *= 0.015; pseudogenes: *W *= 0.0, *P *= 0.015) and non-*Pedicularis* species (CDS genes: *W *= 0.0, *P *= 0.003; pseudogenes: *W *= 52.0, *P *= 0.003).

The synonymous codon usage (RSCU) value for all species is shown in **Supplementary Fig. S3**. Usage was often biased towards one of several codons that encode the same amino acid over the other. For example, the AGA codon had the highest preference for encoding Arginine (R) (RSCU value: 1.7–2.0) among the six synonymous codons. The synonymous codons ending with A or U had higher RSCU values than ending with C or G, such as Tyrosine (Y), Cysteine (C) and Aspartic acid (D).

### Structural variations of the IR boundary

IR regions commonly had seven complete CDS genes (*rpl2, rpl23, ycf2, ycf15, ndhB, rps7, rps12*), six tRNA genes (*trnI-CAU, trnL-CAA, trnV-GAC, trnL-GAU, trnR-ACG, trnN-GUU*), and four rRNA genes (*rrn16, rrn23, rrn4.5, rrn5*). For all species the boundary separating LSC and IRb was between *rps19* and *rpl2* and the boundary separating IRa and LSC was between between *rpl2* and *trnH-GUG.* Boundaries separating IR and SSC regions varied dramatically, especially in section *Cyathophora* (**Fig. 1B**). Few recognize four main types of boundary between these regions. In type I, the *ycf1* gene occurs on the SSC/IRb boundary, with 210–1628 bp located in the IRb region. Type I characterizes all plastomes treated here except for those in section *Cyathophora*. In types II–IV, genes from the SSC have been captured by the IR; all three IR types have captured *ycf1*. In type II plastomes, the IR region includes *ndhH + ndhA *+* ndhF,* characterizing *P. connata, P. cyathophylla, P. cyathophylloides* and *P. rex* var. *rockii*. In type III, the IR region extends to *ndhH *+ *ndhA* alone, characterizing *P. superba*. In type IV, found in series *Reges* except *P. rex* var. *rockii*, includes *ndhH* with *ndhA* inverted in the SSC (**Fig. 1B**). The ancestral state of the *Pedicularis* is type I. A transition to type II occurred in the ancestor of section *Cyathophora*, with types III and IV evolving in the ancestors of clade III and clade I, respectively (**Fig. 1A**).

### Pseudogenization and gene loss in *Pedicularis*

**Fig. 3:**
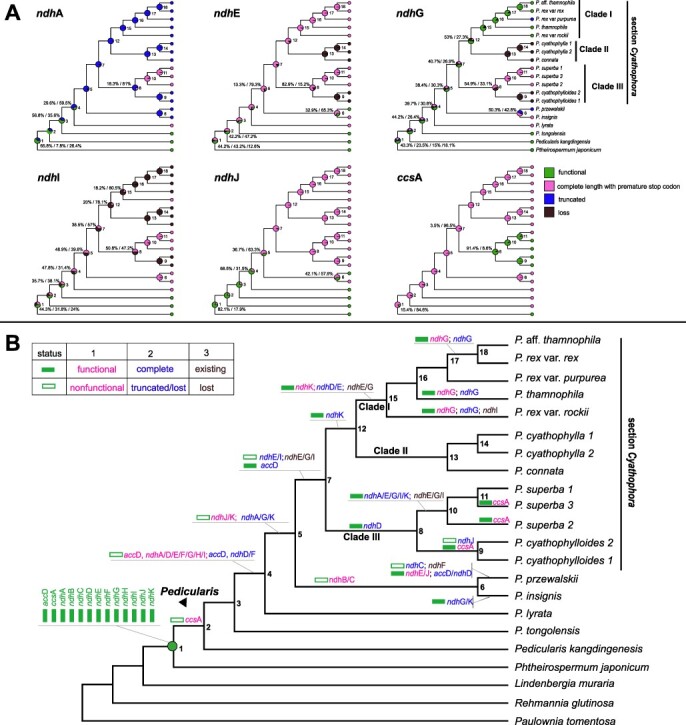
Ancestral state reconstruction of *acc*D, *ccs*A and NDH genes in *Pedicularis* based on the ‘ER’ likelihood model using ‘phytools’ package in R program (A) and parsimony method using Dollop program in the PHYLIP packages (B). Important nodes are annotated by numbers. Genes were classified as four states, i.e. functional (0), full sequence with premature stop codon (1), truncated gene (2) and complete loss (3). In the ‘ER’ model analyses, we inferred ancestral states using four states. In the Dollop analyses, we classified three types: (1) functional (state 0) or non-functional (state 1/2/3), (2) complete (state 0/1) or truncated/lost (state 2/3) and existing (state 0/1/2) or lost (state 3), then we inferred ancestral states using two states.

All plastid genes of *Phtheirospermum, Lindenbergia, Rehmannia* and *Paulownia* were functional. Thirteen pseudogenes were found in *Pedicularis* (**Table 1**, **Figs. 1, 3**), resulting from premature stop codons, deleted 5ʹ- or 3ʹ-untranslated regions, insertions or deletions. The *accD* gene was functional only in *P. tongolensis* and *P. kangdingensis*, having undergone pseudogenization by non-triplet insertions/deletions in other *Pedicularis* species (**Supplementary Fig. S4**). A *ccsA* pseudogene with a small single repeat was found in all *Pedicularis* species, except *P.* c*yathophylloides* and two samples of *P. superba* (2, 3) (**Supplementary Figs. S5, S6**). The number and length of NDH pseudogenes varied, with some persisting as highly degraded fragments (**Table 1**, **Fig. 3**, **Supplementary Fig. S7**). Interestingly, *ndhB* and *ndhC* were pseudogenes only in *P. przewalskii* and *P. insignis*. In *P. insignis, ndhB* only had one mutation [aAt (Leucine) → aCt (stop codon)] and *ndh*C also had one mutation [aAt (Leucine) → aTt (stop codon)] (**Supplementary Fig. S8**). In *P. przewalskii, ndhB* lost four nucleotides and *ndhC* lost the part of the 3ʹ-untranslated region (**Supplementary Fig. S7, S9**). The *ndhA, ndhD, ndhF, ndhH* and *ndhI* genes were functional only in *P. tongolensis* and *P. kangdingensis*. The *ndhE* gene was functional in *P. przewalskii, P. tongolensis* and *P. kangdingensis*. The *ndhG* gene was functional in *P. rex* var. *rex, P. rex* var. *rockii* (**Supplementary Fig. S10**), *P. thamnophila, P. tongolensis* and *P. kangdingensis*. The *ndhJ* gene was functional in *P. przewalskii, P. lyrata, P. tongolensis* and *P. kangdingensis*. The *ndhK* gene was functional in series *Reges, P. lyrata, P. tongolensis* and *P. kangdingensis*.

### Evolutionary selection on plastid genes

Ten of 13 functional gene groups experienced relaxed selection in the hemiparasites and in *Pedicularis* (*k *< 1), although not all these were significant; *cemA* (hemiparasites: *P* > 0.1; *Pedicularis: P* < 0.05) and *rps* (both *P* < 0.05) experienced increased selection (*k* > 1), and *ycf* genes experienced neutral or nearly neutral selection (*k* = 0.98–1.0) (**Table 2**). In section *Cyathophora*, five genes (*cemA, psb, rps, rpo* and *clpP*) showed increased selection, but none of these was significant (*P* > 0.1).

**Table 2:** Results of RELAX analyses for relaxed or intensified selection based on genes concatenated by functional class with hemiparasites, *Pedicularis* and section *Cyathophora* as test group

		Test hemiparasite			Test *Pedicularis*			Test *Pedicularis* sect. *Cyathophora*		
Functional gene group		Relaxation coefficient (k)	*P*-value	Likelihood ratio (LR)	Relaxation coefficient (k)	*P*-value	Likelihood ratio (LR)	Relaxation coefficient (*k*)	*P*-value	Likelihood Ratio (LR)
ATP Synthase	*atp*	0.93	0.427	0.63	0.88	0.172	1.86	0.78	0.248	1.33
Photosynthesis related	*cemA*	1.39	0.138	2.2	1.86	0.019*	5.53	2.86	0.159	1.98
	*pet*	0.75	0.026*	4.99	0.86	0.253	1.31	0.76	0.232	1.43
	*psa*	0.76	0.002**	9.45	0.8	0.009**	6.88	0.51	0.001**	11.56
	*psb*	0.89	0.466	0.53	0.92	0.522	0.41	1.59	0.171	1.87
	*rbcL*	0.47	0.13	2.3	0.38	0.096	2.78	0.24	0.347	0.88
Protein synthesis	*rps*	3.08	0.001**	10.5	2.3	0.029*	4.78	1.47	0.32	0.99
	*rpl*	0.22	0**	21.99	0.22	0**	14.25	0.7	0.726	0.12
	*infA*	0.34	0.029*	4.79	0.26	0.02*	5.43	0.69	0.703	0.14
Plastid-encoded RNA Polymerase	*rpo*	0.87	0.066	3.38	0.76	0**	14.27	1.46	0.248	1.34
‘Other’ functions	*clpP*	0.34	0.2	1.64	0.29	0.306	1.05	2.15	0.304	1.06
	*matK*	0.56	0.648	0.21	0.15	0.272	1.21	0.62	0.754	0.1
	*ycf*	1	0.948	0	0.98	0.627	0.24	0.43	0**	26.45

Significance levels: *P < 0.05,

** *P* < 0.01.

The *ccsA* gene showed increased selection in analyses of the hemiparasitice clade, *Pedicularis* and section *Cyathophora* (*k *> 1.36, *P *< 0.05) (**Table 3**). The *accD* gene showed non-significant relaxation of selection in both hemiparasites and section *Cyathophora* (*k *> 1, *P *> 0.1) and significant intensification of selection (*k *> 1, *P *< 0.05) in *Pedicularis*. The NDH genes showed relaxed selection in both hemiparasites and section *Cyathophora* (*k < *1), except *ndhG* (hemiparasites: *k *= 6.75, *P* < 0.05; and *Pedicularis: k* = 4.46, *P* = 0.062) or *ndhH* [*P* < 0.001 in both hemiparasites (*k* = 6.83) and *Pedicularis* (*k* = 7.32) tests] and *ndhK,* which showed increased selection in *Pedicularis* (*k* = 4.57, *P *= 0.231). Selection was increased for *ndhD* (*k* = 1.66, *P* < 0.001), *ndhG* (*k* = 2.65, *P *= 0.064), *ndh*I (*k* = 2.52, *P *= 0.073), *ndhJ* (*k* = 5.41, *P *= 0.064), and *ndhK* (*k*= 5.53, *P* < 0.01) in section *Cyathophora*.

**Table 3:** Results of RELAX analyses for relaxed or intensified selection based on pseudogenes with hemiparasites, *Pedicularis* and section *Cyathophora* as test group

Gene	Test hemiparasite			Test *Pedicularis*			Test *Pedicularis* sect. *Cyathophora*			Samples*^a^*
	Relaxation coefficient (*k*)	*P*-value	Likelihood ratio (LR)	Relaxation coefficient (*k*)	*P*-value	Likelihood ratio (LR)	Relaxation coefficient (k)	*P*-value	Likelihood ratio (LR)	
*ccsA*	5.76	0**	76.54	1.36	0.013	6.19	3.82	0**	133.9	8/14
*accD*	0.96	0.682	0.17	2.7	0.005**	7.97	0.94	0.86	0.17	6/14
*ndhA*	0.7	0.04*	4.23	0.88	0.839	0.04	0.76	0.017*	5.68	6/4
*ndhB*	0.34	0.214	1.54	0.35	0.206	1.6	0	0.355	0.86	20/2
*ndhC*	0.12	0.006**	7.62	0.09	0.001**	10.19	0	0.105	2.62	20/1
*ndhD*	0.9	0.056	3.66	0.96	0.015*	5.9	1.66	0**	18.2	6/11
*ndhE*	0.18	0**	14.77	0.06	0**	21.31	0	0**	19.62	7/10
*ndhF*	0.4	1	−13.44	0.22	0**	105.43	–	–	–	6/0
*ndhG*	6.72	0.041	4.18	4.46	0.062	3.49	2.65	0.064	3.42	10/5
*ndhH*	6.83	0**	25.04	7.32	0.005**	8.01	0.47	0.681	0.25	6/16
*ndhI*	0.01	0**	16.5	0	0**	14.08	2.52	0.073	3.21	6/6
*ndhJ*	0.5	0.029*	4.75	0.5	0.232	1.43	5.41	0.064	3.43	8/12
*ndhK*	0.99	0.948	0	4.57	0.231	1.43	5.53	0.001**	11.45	12/7

a Number of functional genes/number of genes in full sequence length with premature stop codon. Significance levels:

* *P* < 0.05,

** *P* < 0.01.

### Evolutionary analyses of pseudogenization and gene loss

The ‘ER’ likelihood model analyses showed that the ancestral states of *ndhE*, *ndhG* and *ndhI* may have been pseudogenes in *Pedicularis* (**Supplementary Table S4**). Moreover, both Dollop and the ‘ER’ likelihood model analyses showed that seven NDH genes (*ndhA, ndhD, ndhE, ndhF, ndhG, ndhH* and *ndhI*) lost function at the fourth node (**Fig.****3** and **Supplementary Fig. S11**), and *ndhJ* and *ndhK* lost function at the fifth node (**Fig. 3**). Therefore, nine NDH genes had already lost function in the ancestor of section *Cyathophora.* Based on Dollop and ‘ER’ likelihood model analyses, *ndhI*, which had been previously lost, was regained in *P. superba* and *P. rex* var. *rockii*, and the truncated *ndhA* recovered its full length in the *P. superba* clade (**Fig. 3**). The Dollop analysis showed that the functional loss of *ndhK* happened at the fifth node and, then, reversed to full function in clade I of section *Cyathophora* (**Fig. 3B**), but the ‘ER’ likelihood model analysis showed *ndhK* underwent no functional reversal but rather experienced three independent functional losses at the sixth node, and in clades II and III of section *Cyathophora* (**Supplementary Fig. S11**). The Dollop analysis showed that *ndhG* lost function at the fourth node, followed by three reversals in series *Reges* except *P. rex* var. *purpurea* (**Fig. 3B**). However, the ‘ER’ likelihood model analysis showed that *ndhG* had no significantly functional reversals with independent fragmentation and physical loss (**Fig. 3A**).

Both analyses showed that the functional loss of *ccs*A occurred in the basal branches of *Pediculari*s (**Fig. 3** and **Supplementary Table S4**). This was followed by a reversal to full function in the eighth node (91.4% support) of clade III and a second loss of function in *P. superba* 1 (**Fig. 3**). Both analyses showed that *accD* lost function at the fourth node (**Fig.****3** and **Supplementary Fig. S11**). Dollop analysis showed that *accD* regained its full length in *P. przewalskii* at the seventh node (**Fig.****3B**).

## Discussion

### Plastid phylogenomics of section *Cyathophora*

Plastid phylogenomics recovered the monophyly of section *Cyathophora* as reported in previous studies (e.g. Eaton and Ree 2013, Yu et al. 2013, 2015, Wang et al. 2017). Monophyly of series *Reges* was also recovered. The relationship among the other three series has been controversial using nuclear and plastid datasets (Eaton and Ree 2013, Yu et al. 2013, 2015, Wang et al. 2015, 2017). Phylogenies based on RAD-seq, transcriptome, *CRABS CLAW* and nrITS dastasets supported a well-supported clade including *P. cyathophylla, P. cyathophylloides* and *P. superba*. However, phylogenies based on *LEAFY* and plastid data provided poor and conflicting resolution among these species. In this study, plastid phylogenomics strongly supported a clade composed of *P. cyathophylloides *+* P. superba* and a different clade composed of *P. cyathophylla* + *P. connata*. The *P. cyathophylloides *+* P. superba* clade was the first diverging clade in section *Cyathophora*, as sister to the *P. cyathophylla* + *P. connata* clade and series *Reges*. Noteworthily, *P. connata* is included in phylogenetic analyses for the first time and is sister to *P. cyathophylla*, corresponding to series *Cyathophyllae* in the classification system of Li (1948).

### Plastome characteristics of *Pedicularis*

In this study, the plastomes of hemiparasitic *Pedicularis* and the outgroups have the typical quadripartite architecture, but the IR/SSC boundaries show variation in the recently derived section *Cyathophora*. Generally, the plastome structure and gene contents are highly conserved at the genus level or in recently derived lineages. IR regions in section *Cyathophora* can be classified into three types. These types are associated with the species delimitation supported by plastid phylogenies (Yu et al. 2013, Wang et al. 2015), with the exception in series *Reges* of *P.* rex var. *rockii*, in which the IR region differed from other varieties of *P. rex* (**Fig. 1**). IR regions may have experienced a large expansion of the SSC region in the common ancestor of section *Cyathophora*, followed by two independent contractions, in *P. superba* and series *Reges*. The expansions and contractions of the IR regions in section *Cyathophora* might have destabilized the plastome. This, along with changes in selective pressures on the NDH and *ccsA* genes located at the junctions between the IR and SSC regions, may have caused more frequent parallel losses or reversals of these genes as opposed to others in the LSC and IR regions (**Fig. 3**).

IR expansion in section *Cyathophora* (**Table 1**, **Fig. 1**) increased the length of the plastome despite gene fragmentation. It is worth noting that the sizes of the IR and SSC correlate with their GC contents, possibly due to IR capture of SSC sequences. The GC content of NDH genes (except *ndhC, ndhJ* and *ndhK*) in section *Cyathophora* (35.3 to 36.9%) was lower than that of the IR (40.5 to 41.1%). Consequently, the GC content of the IR expanded region in section *Cyathophora* was significantly lower than that of both other *Pedicularis* and non-*Pedicularis*. IR expansion may have altered the structural conservatism of the plastome (Sabir et al. 2014, Zhu et al. 2016).

The size of the LSC was negatively correlated with its own GC content (**Fig. 2**). Due to the expansion of the IR and reduction of GC content in section *Cyathophora*, whole plastome size is negatively correlated with the total GC content. Therefore, the total GC content of the section *Cyathophora* was lower than that of other *Pedicularis*. Interestingly, the average GC content of *Pedicularis* (38.2–38.6%) and *Phtheirospermum japonicum* (38.3%) were higher than those of three closely related autotrophs (37.7–38.0%) (**Table 1**), suggesting that the GC content might be increased or not significantly decreased in the fully photosynthetic hemiparasitic plants in an early stage of the plastome degradation. Some studies have demonstrated that the shift from non-parasite to parasite is accompanied by reduction in plastome GC content, especially in holoheterotrophic plants (Wicke and Naumann 2018, Su et al. 2019).

### Pseudogenization and loss of NA(D)H dehydrogenase-like genes in *Pedicularis*

The 11 NDH genes were pseudogenized or completely lost in the plastomes of *Pedicularis* (**Fig. 3**), as has been observed in other members of Orobanchaceae (e.g. Wicke et al. 2013, Frailey et al. 2018). Based on the comprehensive phylogeny of *Pedicularis* (Yu et al. 2015), the two species in the basal clades, *P. kangdingensis* and *P. tongolensis*, have functional NDH genes, as well as the sister lineage *Phtheirospermum japonicum*. Ancestral state reconstruction indicated that the common ancestor of *Pedicularis* had functional NDH genes, with subsequent pseudogenization and loss (**Table 3**). This evolutionary pattern indicated that the degradation of the NDH genes in *Pedicularis* was a gradual process from functional to non-functional. Moreover, gene loss and pseudogenization of NDH genes likely occurred independently across the 13 major clades in *Pedicularis* (Zhang et al. 2020), which is similar to the pattern of evolution proposed for vegetative and corolla characters (Ree 2005, Yu et al. 2015).

In photosystem II, the NDH proteins act by adjusting the redox level of the cyclic electron transport machineries, allowing the fine tuning of photosynthesis (Peltier et al. 2016, Shikanai 2016, Laughlin et al. 2019). The mechanism of NDH gene loss remains unclear, and published studies have suggested that some NDH genes might be dispensable or their lost function compensated by other factors (Suorsa et al. 2012, Strand et al. 2019). Functional and/or physical loss of NDH genes occurs in some non-parasitic plants, including gymnosperms (Braukmann et al. 2009), Geraniaceae (Blazier et al. 2011) and Lentibulariaceae (Wicke et al. 2014), besides heterotrophic plants (Graham et al. 2017, Wicke and Naumann 2018). Thus, the loss of NDH genes might be a random phenomenon in photosynthetic lineages, though common in the heterotrophic plants (Wicke and Naumann 2018). In mycoheterotrophic orchid lineages (including leafy and photosynthetic orchids), NDH gene loss may have not been so disadvantageous for the lineages that live in low-light canopy habitats as epiphytes, or in dark, understory habitats (Barrett et al. 2014, 2019, Feng et al. 2016). In parasitic plants, loss of NDH genes may be associated with increasing dependence on host-derived carbon and decreasing dependence upon photosynthetic carbon (Wicke et al. 2016, Wicke and Naumann 2018). For example, Bao (2020) found that the net photosynthetic rate of *Pedicularis kansuensis* Maxim decreased significantly in plants grown on hosts rather than those without hosts.

### Pseudogenization of *accD* and *ccsA* genes

The plastid *accD* gene encodes a beta subunit of Acetyl-CoA carboxylase (ACCase), which is a key enzyme for fatty acid biosynthesis and is crucial for leaf development (Kode et al. 2005, Bock 2007). Functional loss of *accD* has occurred in autotrophic angiosperms (Sasaki and Nagano 2004, Rousseau-Gueutin et al. 2013, Ruhlman and Jansen 2014) and gymnosperms (Sudianto and Chaw 2019), as well as Volvocales (Smith and Lee 2014), in which the function might be taken over by the nuclear copy (Sasaki and Nagano 2004, Wicke et al. 2011). The acc*D* gene maintains intact reading frames in most heterotrophic plants (Wicke and Naumann 2018). In this study, the *accD* is highly divergent in *Pedicularis*, with long insertions/deletions and high rates of substitution. Function of *accD* may be taken over by nuclear copies (Sasaki and Nagano 2004, Rousseau-Gueutin et al. 2013, Sudianto and Chaw 2019), because nuclear copies of an *acc*D-like gene were isolated from transcriptome data of *Pedicularis* (**Supplementary Fig. S12**).

The *ccs*A gene encodes a cytochrome C biogenesis protein that mediates the attachment of heme to c-type cytochromes (Bock 2007, Ruhlman and Jansen 2014). This gene is localized in the plastid SSC region and functional in most photosynthetic plants (Wicke et al. 2011). However, it is lost or pseudogenized in most heterotrophic plants (Graham et al. 2017, Wicke and Naumann 2018). In *Pedicularis*, the ccsA gene is not truncated but has experienced frameshift in most species resulting in a premature stop codon (**Supplementary Fig. S5, S6**). Exceptions include members of clade III of section *Cyathophora* which have functional *ccsA* genes (**Fig. 3B**). Two of three individuals of *P. superba* had functional *ccsA*. This polymorphism might be recovered in other species of *Pedicularis* if more individuals are genotyped in the future. Unexpectedly, evolutionary reconstruction of the ancestral state suggests that there may have been one or three reversals from non-functional to functional in *P. cyathophylloides* and *P. superba* (**Fig. 3**). The RELAX (Detecting Relaxed Selection) analysis indicated that the *ccs*A gene experienced increased selection in the hemiparasites, *Pedicularis*, and section *Cyathophora*, which suggests that *ccsA* gene function is necessary in these lineages.

### Evolutionary analyses of plastid genes

Relaxed selective pressure on plastid genes is one important driver of plastome evolution in heterotrophic plants (Wicke et al. 2016). The RELAX selection analyses detected relaxed selection in 11 functional group genes (**Table 2**) and up to nine NDH genes in hemiparasitic *Pedicularis* (**Table 3**), which might have caused variation in the acc*D* and NDH genes in *Pedicularis*, even within a species. We detected significant relaxation of selection on fully functional photosynthesis and housekeeping genes in *Pedicularis*. Relaxation of selective pressure on photosynthesis and housekeeping genes has also been found in other heterotrophic plants, including mistletoes (Chen et al. 2019), *Cuscuta* (Banerjee and Stefanovic 2019), Orobanchaceae (Wicke et al. 2016, Frailey et al. 2018) and Orchidaceae (Barrett et al. 2014, 2019, Feng et al. 2016), which also show variation in *accD, ccsA* and NDH genes. However, this study is the first to report similar variation in acc*D*, ccs*A* and NDH genes in a recently derived lineage, section *Cyathophora*, which diverged only 7.14 Mya (Wang et al. 2015). As discussed above, the full functions of the *accD*, ccs*A* and NDH genes in the plastome are not clear, but these genes normally play critical roles in photosynthesis in green plants. The relaxed selection on these genes is an important indicator for plants transition from non-parasite to parasite (Wicke et al. 2016). The question remains, however, how hemiparasitic plants carry on photosynthesis without functional plastid *ccsA* and NDH genes. Of course, hemiparasites can use haustoria to steal nutrients from the host plants to compensate the loss of the function of these genes (Wicke et al. 2016, Yoshida et al. 2016, Wicke and Naumann 2018) and perhaps survive as well with less efficient photosynthetic apparatus.

It is interesting that some photosynthesis-related genes showed increased selection in the RELAX selection tests. Of these, *cemA, ccsA* and *ndhG* showed consistent intensification in the three tests, while others experienced increased selection pressure in section *Cyathophora*. The functional *ccsA* gene in section *Cyathophora* is presumably associated with the intensification of selection detected in these genes.

## Material and Methods

### Plant materials, DNA extraction and sequencing

In total, 22 new plastomes were de novo assembled and analyzed in this study (**Supplementary Table S2**). Of these, 13 samples represent the six recognized species of *Pedicularis* sect. *Cyathophora* (including one individual each of *P. connata* and *P. cyathophylla*, two of *P. thamnophila* and three of *P. cyathophylloides, P. rex* and *P. superba*), and five represent selected *Pedicularis* species, including two species (*P. insignis* and *P. przewalskii*) sister to section *Cyathophora*, and *P. kangdingensis, P. lyrata* and *P. tongolensis*, representing three recognized clades of *Pedicularis* (Yu et al. 2015). The hemiparasitic *Phtheirospermum japonicum* and the autotrophic *Lindenbergia muraria* (Orobanchaceae), *Rehmannia glutinosa* (Orobanchaceae) and *Paulownia tomentosa* (Paulowniaceae) were sampled as outgroups. Voucher specimens of 20 newly sequenced taxa were deposited in the herbarium of Kunming Institute of Botany, Chinese Academy of Science (KUN). The raw reads of *Phtheirospermum japonicum* (DRR082673) (Kado and Innan 2018) and *Paulownia tomentosa* (SRR6940033) (Mint Evolutionary Genomics Consortium 2018) were downloaded from the Sequence Read Archive (SRA) data of NCBI.

Total genomic DNA was extracted from silica gel-dried leaf tissues or nitrogen-frozen young buds using a modified CTAB method. Purified DNA was fragmented to approximately 500 bp in size for library construction following standard protocols (NEBNext® Ultra II™DNA Library Prep Kit for Illumina®). The 150 bp pair-end reads were generated using the Illumina Hi-Seq 2500.

### Plastome assembly and annotation

Circular plastomes were de novo assembled using the GetOrganelle toolkit (Jin et al. 2020). The final circular plastid graphs were also checked using Bandage (Wick et al. 2015). The quality of the plastome assemblies was assessed by reads mapping using the GetOrganelle toolkit. Plastomes of four non-*Pedicularis* species and *P. tongolensis* were automatically annotated using CPGAVAS2 (Shi et al. 2019) and, then, manually adjusted in Geneious (Biomatters, Auckland, New Zealand) using *Nicotiana tabacum* L. (accession number: Z00044) as the reference. The remaining plastomes of *Pedicularis* were manually annotated by Geneious using *P. tongolensis* as the reference. Fragmented genes were checked and confirmed using BLAST. The plastome maps were drawn by Organellar Genome DRAW tool (Greiner et al. 2019).

### Statistical analysis of plastomes

We analyzed the relative synonymous codon usage (RSCU) value using MEGA-X (Kumar et al. 2018) and then drew the heatmap in the R statistical environment. RSCU > 1: codons used more frequently than expected; RSCU = 1: codons used as frequently as expected; RSCU < 1: codons used less frequently than expected. We used Spearman’s rank correlation (Best and Roberts 1975) for correlation analyses and the Wilcoxon test (Bauer 1972) for difference comparisons among genomic characters (i.e. GC content, genome size, etc.) in the R statistical environment.

### Phylogenomic analysis

The whole sequence of 22 plastomes with one IR region was aligned using MAFFT (Katoh and Standley 2013) using the default options and then manually adjusted in Geneious. The aligned matrix was used to reconstruct the phylogeny using the maximum likelihood (ML) method. ML analysis was conducted using RAxML (Stamatakis et al. 2008), with GTR + GAMMA + I model to find the best-scoring ML tree. One thousand bootstrap replicates were performed to obtain clade support. ML analysis was performed at the CIPRES Science Gateway (http://www.phylo.org). The tree was viewed and edited with FigTree (http://tree.bio.ed.ac.uk/software/figtree/).

### Testing selection of plastid genes

First, we used MACSE v2 (Ranwez et al. 2011) to align CDS regions with pseudogenes. Then, we aligned functional CDS genes in Geneious using the Translation Align option. The alignments for each CDS gene and 13 functional gene groups (see **Table 2**) were used to test for potential relaxed selection on the Datamonkey Adaptive Evolution Server (https://www.datamonkey.org/) with the HyPhy software package (Pond and Muse 2005) using the hypothesis testing framework RELAX (Wertheim et al. 2014). We examined three test groups, i.e. hemiparasites (vs. non-parasites), *Pedicularis* (vs. non-*Pedicularis*) and *Pedicularis* sect. *Cyathophora* (vs. others).

RELAX calculates the ratio of nonsynonymous (*dN*) to synonymous (*dS*) substitutions. Given two subsets of branches in a phylogeny, RELAX can determine whether selective strength was relaxed or intensified in one of these subsets relative to the other. Rate changes are summarized using a relaxation coefficient (*k*), where *k *< 1 indicates the relaxation of selection and *k *> 1 indicates the intensification of selection.

### Evolutionary analyses of IR structure, pseudogenization and gene loss

Because the IR regions showed dramatic variations in *Pedicularis*, especially in *Cyathophora*, four types of IR structure were recognized (see the ‘Results’ section). To infer the evolutionary history of the four types, ancestral states were reconstructed based on the plastome ML tree using the ‘phytools’ (phylogenetic tools for comparative biology—and other things) package (Revell 2012) in the R statistical environment with the ‘ER’ likelihood model (Schluter et al. 1997).

The plastome tree was also used to infer ancestral states of the 11 NDH genes, *accD* and *ccsA*, with functional and physical losses inferred using the ‘phytools’ package (Revell 2012) and the Dollop program of PHYLIP (http://evolution.genetics.washington.edu/phylip.html), respectively. The four states for each gene specified were functional (0, an ancestral state), full sequence with premature stop codon (1), truncated gene (2) and complete loss (3). First, the ancestral state for each gene was estimated by Brownian evolution using likelihood with the ‘ER’ model (Schluter et al. 1997). We then used the Dollo parsimony method to infer the evolutionary history of the 13 genes. The ancestral state (0) was known in each gene. Once state 1 was reached, the reoccurrence of state 0 is very improbable, much less probable than multiple retentions of polymorphism. For the Dollop parsimony analyses, we specified a transition matrix allowing changes from functional (0) → nonfunctional (1,2,3), complete (0,1) → truncated or lost (2,3) and existing (0,1,2) → complete loss (3), with equal probabilities, and restricted all reversals.

## Supplementary Material

pcab074_Supp

## Data Availability

The data underlying this article are available in the GenBank Nucleotide Database at https://www.ncbi.nlm.nih.gov/ and can be accessed with accession numbers MZ264869-MZ264890. The data were also deposited at figshare with the DOI https://doi.org/10.6084/m9.figshare.14633430.
