# Supplementary material for: Plastid NDH Pseudogenization and Gene Loss in a Recently Derived Lineage from the Largest Hemiparasitic Plant Genus Pedicularis (Orobanchaceae)
Source: Plant Cell Physiol. 2021 May 28;62(6):971–84. doi: 10.1093/pcp/pcab074 (PMC8504446; doi:10.1093/pcp/pcab074)
Supplement: pcab074_Supp [file pcab074_supp.zip › pcp-2021-e-00042-File005.pdf]

Supplementary data

Dramatic variations in plastid NDH pseudogenization and gene loss in a recently derived hemiparasitic lineage

Xin Li[1,2,5], Jun-Bo Yang[3], Hong Wang[4], Yu Song[1,2,6], Richard T. Corlett[1,2], Xin Yao[1,2], De-Zhu Li[3*], Wen-Bin Yu[1,2,6 *]

1 *Center for Integrative Conservation, Xishuangbanna Tropical Botanical Garden, Chinese Academy of Sciences, Mengla, Yunnan 666303, China*
2 *Center of Conservation Biology, Core Botanical Gardens, Chinese Academy of Sciences, Mengla, Yunnan 666303, China*
3 *Plant Germplasm and Genomics Center, Germplasm Bank of Wild Species, Kunming Institute of Botany, Chinese Academy of Sciences, Kunming, Yunnan 650201, China*
4 *Key Laboratory for Plant Diversity and Biogeography of East Asia, Kunming Institute of Botany, Chinese Academy of Sciences, Kunming, Yunnan 650201, China*
5 *University of Chinese Academy of Sciences, Shijingshan District, Beijing 100049, China*
6 *Southeast Asia Biodiversity Research Institute, Chinese Academy of Science, Yezin, Nay Pyi Taw 05282, Myanmar*

*, Corresponding authors: De-Zhu Li (dzl@mail.kib.ac.cn); Wen-Bin Yu (yuwenbin@xtbg.ac.cn),

ORCID:

Wen-Bin Yu (https://orcid.org/0000-0002-7643-2112)

De-Zhu Li (https://orcid.org/0000-0002-4990-724X)

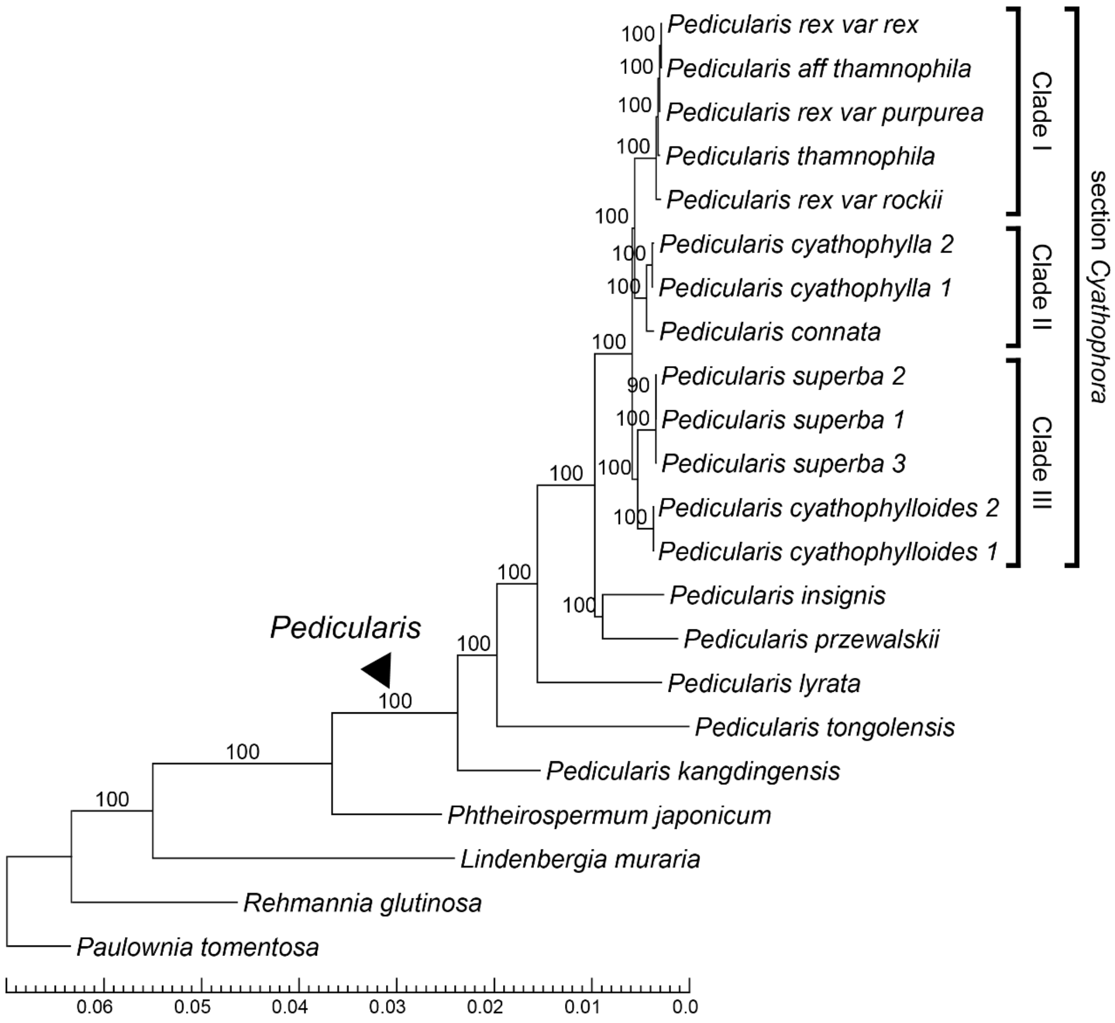

Figure S1. Phylogenetic relationship of *Pedicularis* sect. *Cyathophora* inferred by Maximum Likelihood (ML) approach using the whole plastome data with one IR region. ML Bootstrap values are presented above branches. The bottom scale bar represents the number of substitutions per site.

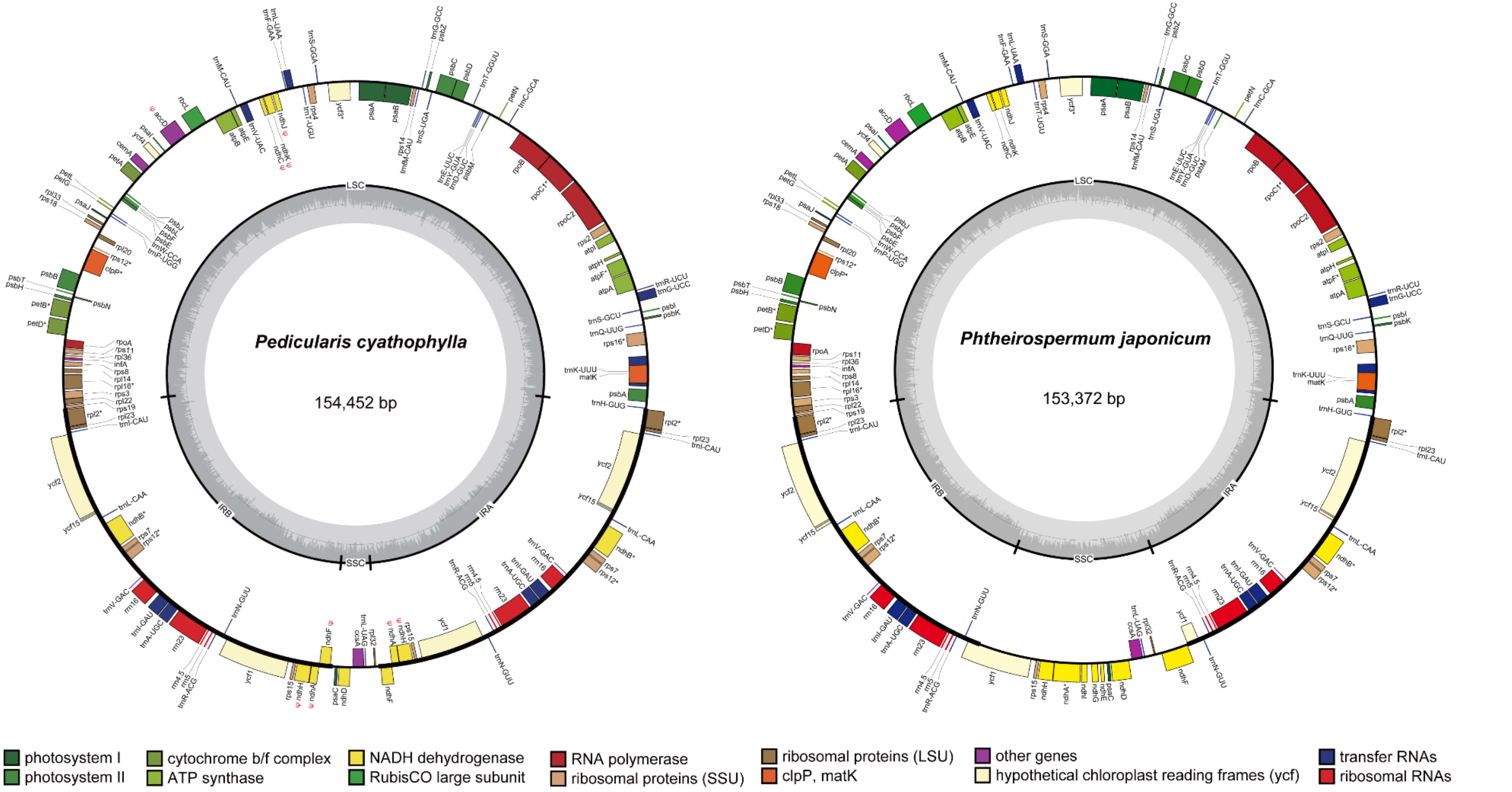

Figure S2. Circular map of the plastome of *Pedicularis cyathophylla* and *Phtheirospermum japonicum*. Genes shown outside the outer circle are transcribed clockwise, while inside genes are counterclockwise. Pseudogenes are marked by "ψ" in red. Drawing made using OGDRAW v.1.3.1.

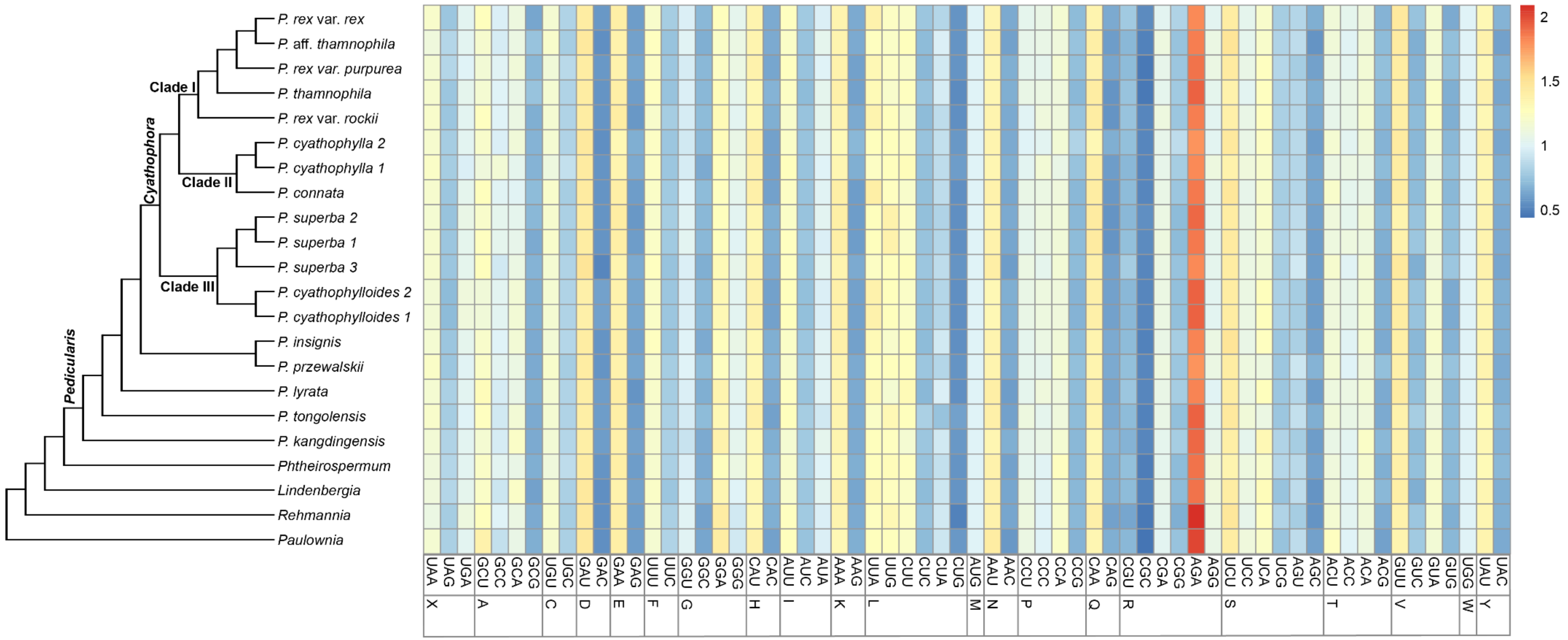

Figure S3. Heatmap of codon usage of 20 amino acids and stop codon in 80 coding regions (right). The species was ordered using the plastid phylogenomic tree (see Supplementary Figure S1).

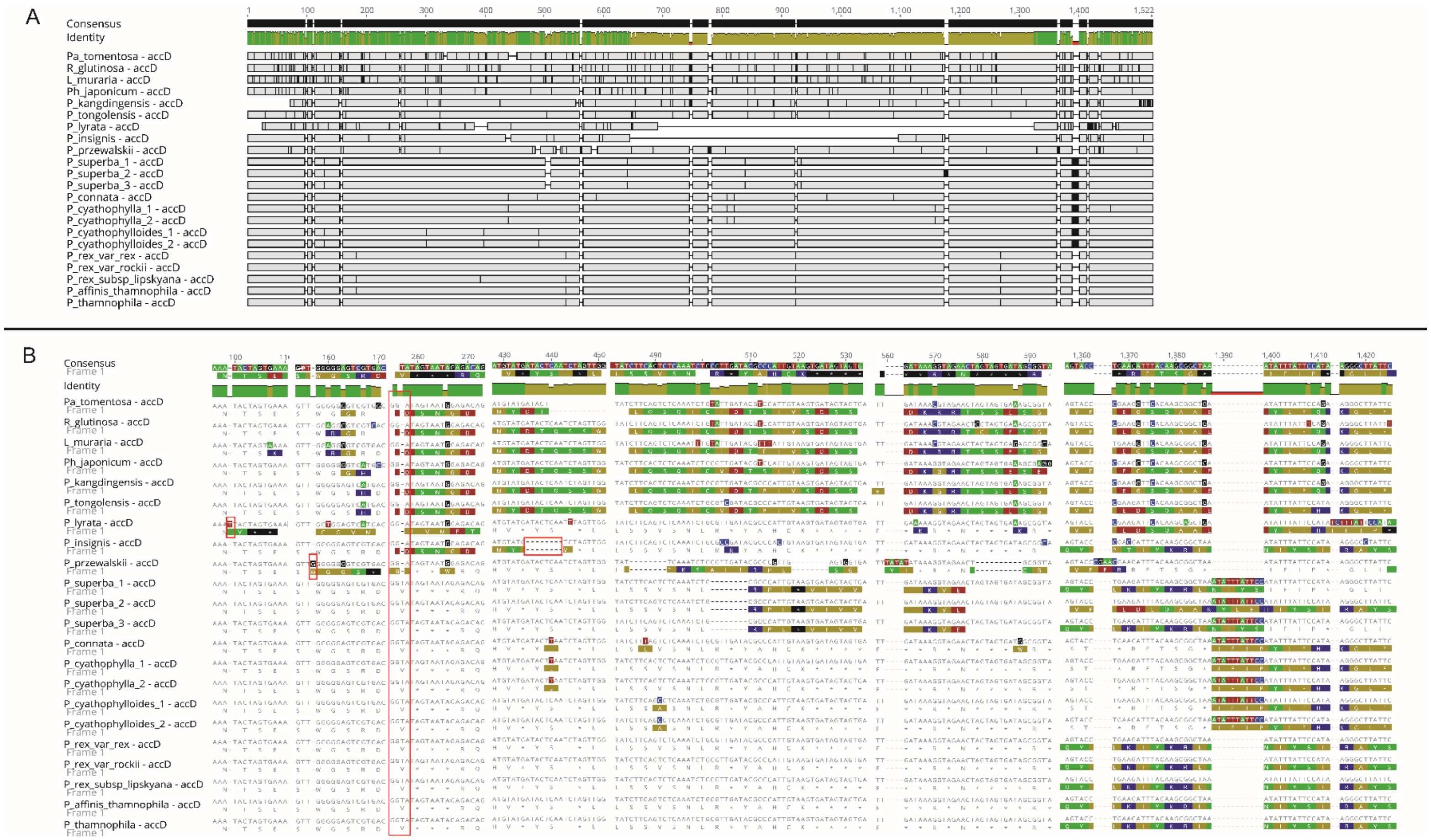

Figure S4. The overall view of *acc*D gene alignment in 22 species (A). The consensus identity: the green color representing all sequences in columns have identical nucleotides, and the yellow color representing one or multiple sequences in columns have different nucleotides by mutations or short insertions/deletions, and the red color or line representing some sequences in columns have long insertions/deletions. Some selected regions for mutations and deletion/insertions in the *acc*D gene alignment (B).

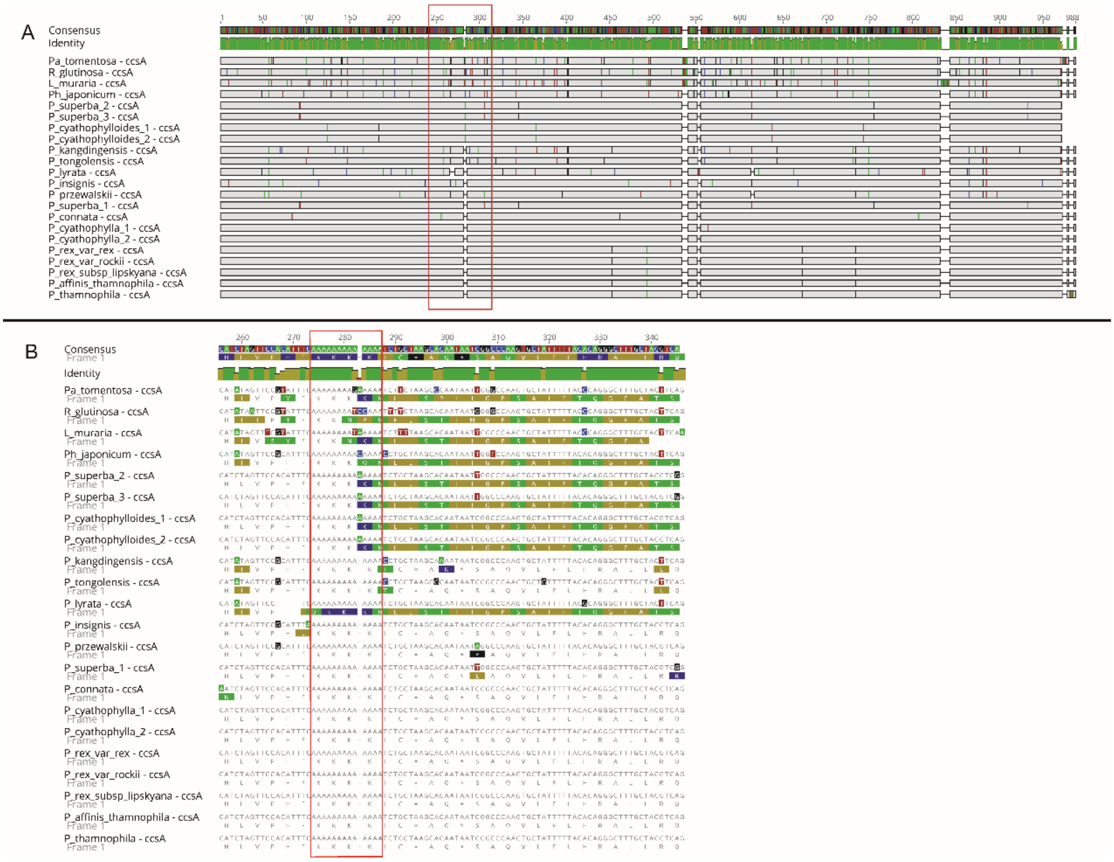

Figure S5. The overall view of *ccs*A gene alignment in 22 species (A). The consensus identity: the green color representing all sequences in columns have identical nucleotides, and the yellow color representing one or multiple sequences in columns have different nucleotides by mutations or short insertions/deletions, and the red color or line representing some sequences in columns have long insertions/deletions. The selected region showed a nucleotide indels in a mononucleotide repeat region of the *ccs*A gene (B).

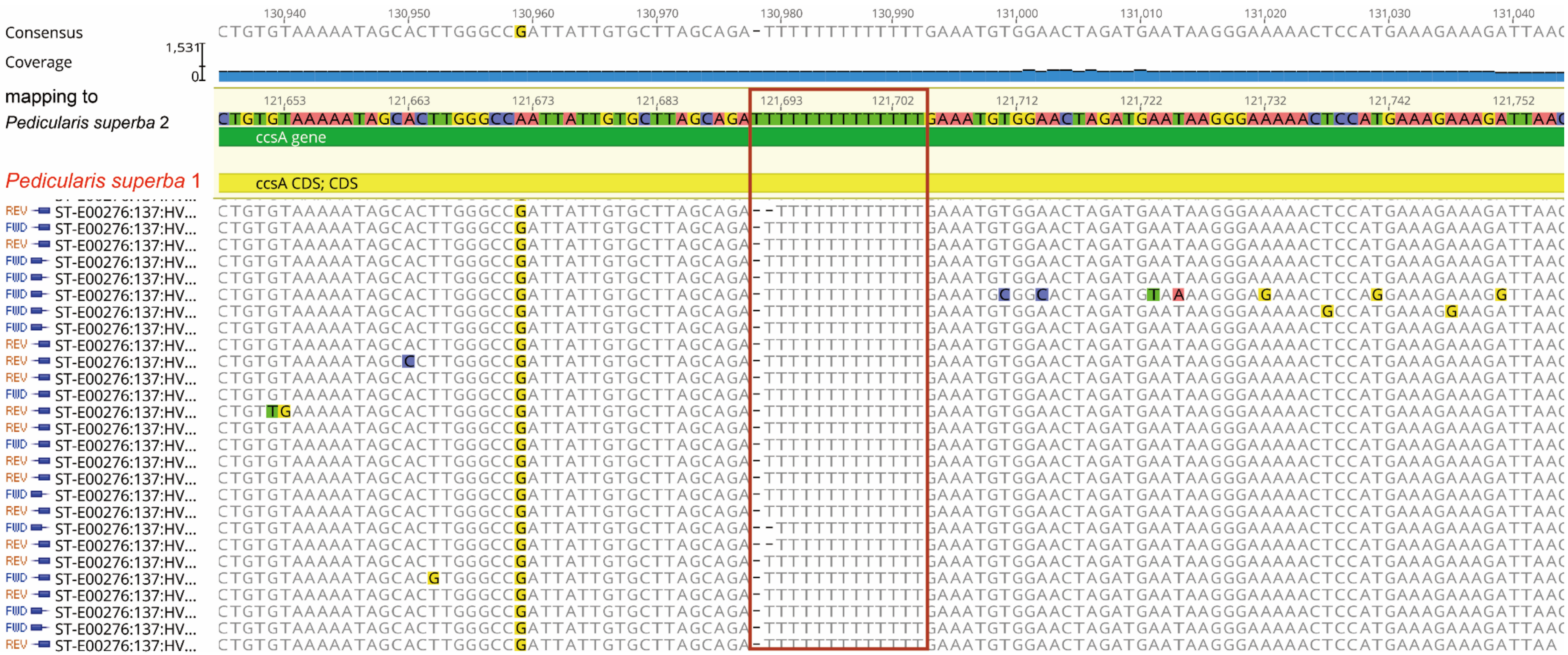

Figure S6. Raw reads of *Pedicularis superba* (1) mapping to the plastome *P. superba* (2) as the reference in the *ccs*A gene region. The raw reads of *P. superba* (1) have one (or two) base deletion in a mononucleotide repeat region of the *ccs*A gene.

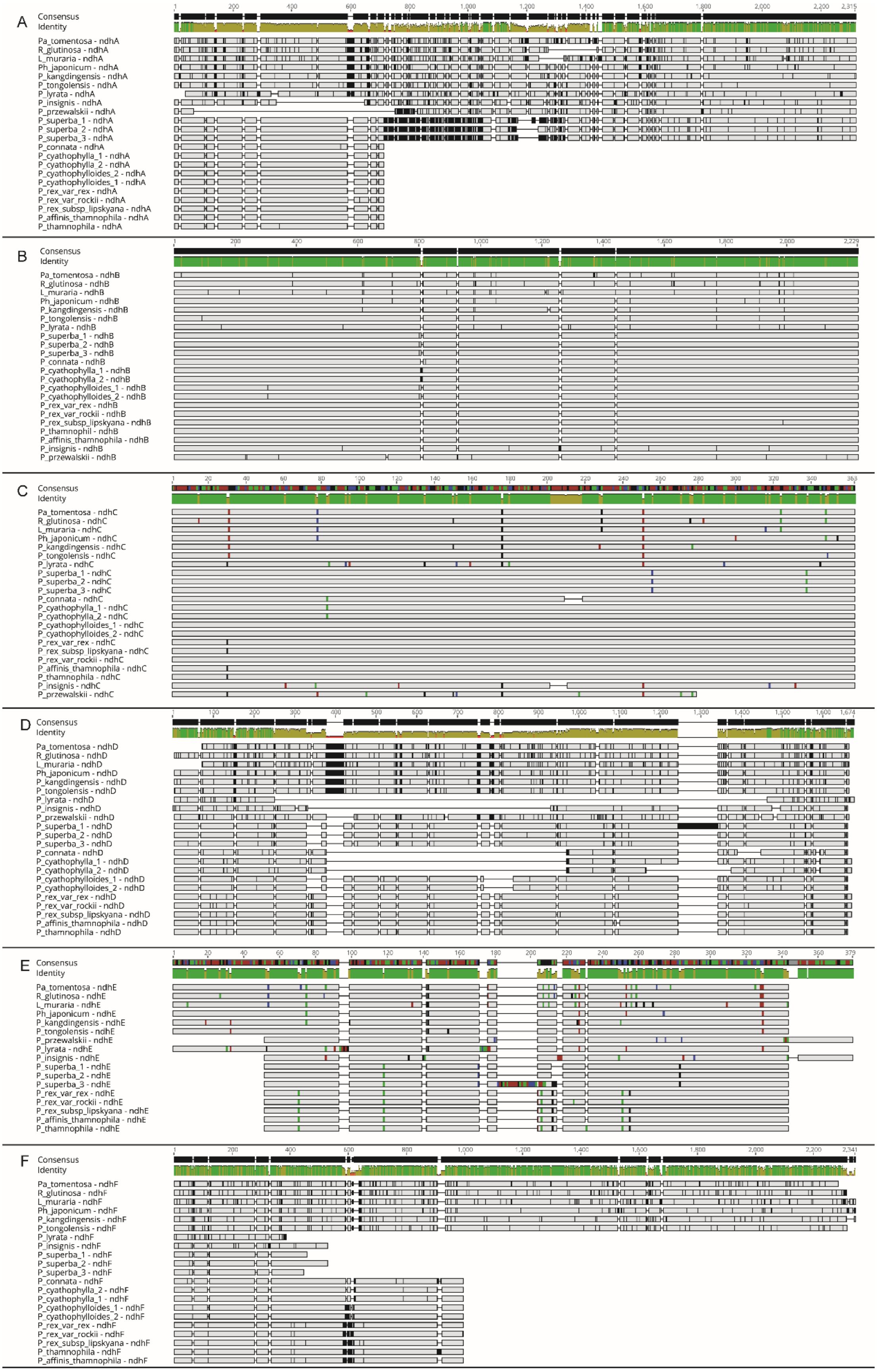
A
Consensus
Identity
Pa_tomentosa - ndhA
R_glutinosa - ndhA
L_muraria - ndhA
Ph_japonicum - ndhA
P_kangdingensis - ndhA
P_tongolensis - ndhA
P_lyrata - ndhA
P_insignis - ndhA
P_przewalskii - ndhA
P_superba_1 - ndhA
P_superba_2 - ndhA
P_superba_3 - ndhA
P_connata - ndhA
P_cyathophylla_1 - ndhA
P_cyathophylla_2 - ndhA
P_cyathophylloides_2 - ndhA
P_cyathophylloides_1 - ndhA
P_rex_var_rex - ndhA
P_rex_var_rockii - ndhA
P_rex_subsp_lipskyana - ndhA
P_affinis_thamnophila - ndhA
P_thamnophila - ndhA
B
Consensus
Identity
Pa_tomentosa - ndhB
R_glutinosa - ndhB
L_muraria - ndhB
Ph_japonicum - ndhB
P_kangdingensis - ndhB
P_tongolensis - ndhB
P_lyrata - ndhB
P_superba_1 - ndhB
P_superba_2 - ndhB
P_superba_3 - ndhB
P_connata - ndhB
P_cyathophylla_1 - ndhB
P_cyathophylla_2 - ndhB
P_cyathophylloides_1 - ndhB
P_cyathophylloides_2 - ndhB
P_rex_var_rex - ndhB
P_rex_var_rockii - ndhB
P_rex_subsp_lipskyana - ndhB
P_thamnophil - ndhB
P_affinis_thamnophila - ndhB
P_insignis - ndhB
P_przewalskii - ndhB
C
Consensus
Identity
Pa_tomentosa - ndhC
R_glutinosa - ndhC
L_muraria - ndhC
Ph_japonicum - ndhC
P_kangdingensis - ndhC
P_tongolensis - ndhC
P_lyrata - ndhC
P_superba_1 - ndhC
P_superba_2 - ndhC
P_superba_3 - ndhC
P_connata - ndhC
P_cyathophylla_1 - ndhC
P_cyathophylla_2 - ndhC
P_cyathophylloides_1 - ndhC
P_cyathophylloides_2 - ndhC
P_rex_var_rex - ndhC
P_rex_subsp_lipskyana - ndhC
P_rex_var_rockii - ndhC
P_affinis_thamnophila - ndhC
P_thamnophila - ndhC
P_insignis - ndhC
P_przewalskii - ndhC
D
Consensus
Identity
Pa_tomentosa - ndhD
R_glutinosa - ndhD
L_muraria - ndhD
Ph_japonicum - ndhD
P_kangdingensis - ndhD
P_tongolensis - ndhD
P_lyrata - ndhD
P_insignis - ndhD
P_przewalskii - ndhD
P_superba_1 - ndhD
P_superba_2 - ndhD
P_superba_3 - ndhD
P_connata - ndhD
P_cyathophylla_1 - ndhD
P_cyathophylla_2 - ndhD
P_cyathophylloides_1 - ndhD
P_cyathophylloides_2 - ndhD
P_rex_var_rex - ndhD
P_rex_var_rockii - ndhD
P_rex_subsp_lipskyana - ndhD
P_affinis_thamnophila - ndhD
P_thamnophila - ndhD
E
Consensus
Identity
Pa_tomentosa - ndhE
R_glutinosa - ndhE
L_muraria - ndhE
Ph_japonicum - ndhE
P_kangdingensis - ndhE
P_tongolensis - ndhE
P_przewalskii - ndhE
P_lyrata - ndhE
P_insignis - ndhE
P_superba_1 - ndhE
P_superba_2 - ndhE
P_superba_3 - ndhE
P_rex_var_rex - ndhE
P_rex_var_rockii - ndhE
P_rex_subsp_lipskyana - ndhE
P_affinis_thamnophila - ndhE
P_thamnophila - ndhE
F
Consensus
Identity
Pa_tomentosa - ndhF
R_glutinosa - ndhF
L_muraria - ndhF
Ph_japonicum - ndhF
P_kangdingensis - ndhF
P_tongolensis - ndhF
P_lyrata - ndhF
P_insignis - ndhF
P_superba_1 - ndhF
P_superba_2 - ndhF
P_superba_3 - ndhF
P_connata - ndhF
P_cyathophylla_2 - ndhF
P_cyathophylla_1 - ndhF
P_cyathophylloides_1 - ndhF
P_cyathophylloides_2 - ndhF
P_rex_var_rex - ndhF
P_rex_var_rockii - ndhF
P_rex_subsp_lipskyana - ndhF
P_thamnophila - ndhF
P_affinis_thamnophila - ndhF

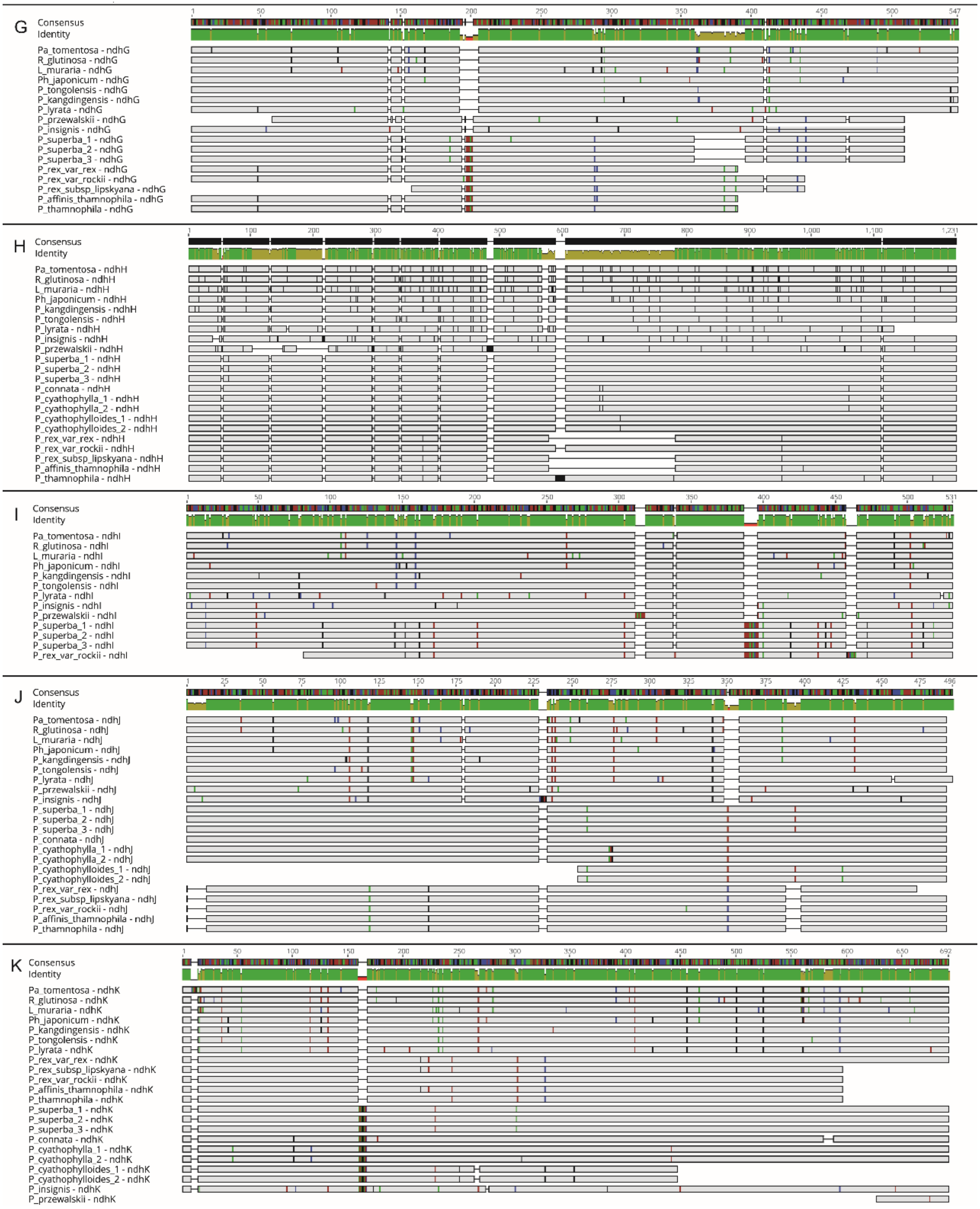

Figure S7. The overall situation of NDH (A-K) genes regions alignment in 22 species. The consensus identity: the green color means all sequence code were consistent, the yellow color means some sequence code were inconsistent (mutation or insertion), and the red color or line means some sequence were lost.

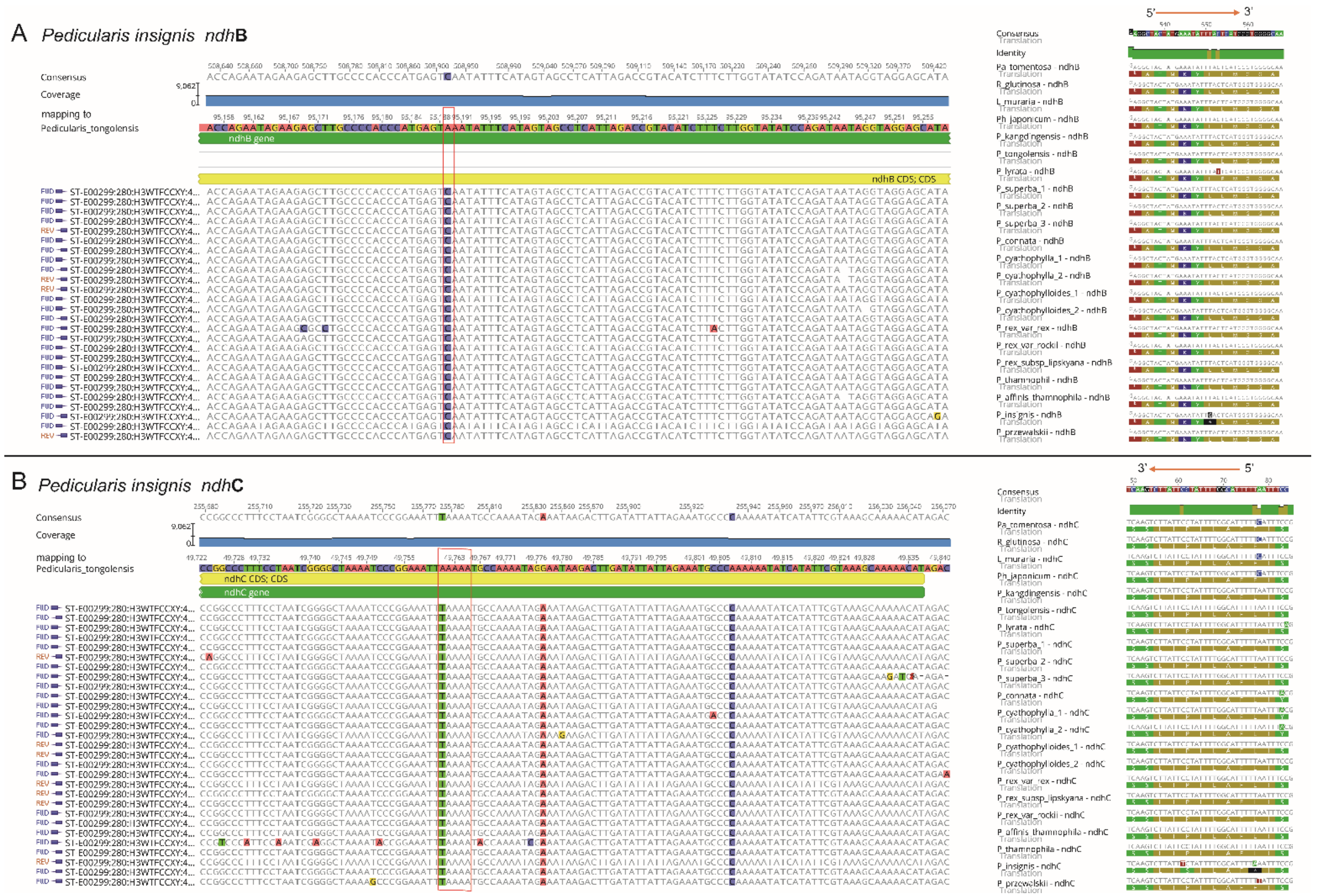

Figure S8. Raw reads of *Pedicularis insignis* mapping to the plastome of *P. tongolensis* as the reference in *ndh*B gene (A) and *ndh*C gene (B). The top one (A) showing the raw reads of *P. insignis* had one base changed from A to C (red box) in the *ndh*B gene, and the bottom one (B) showing the raw reads of *P. insignis* had one base changed from A to T (red box) in the *ndh*C gene.

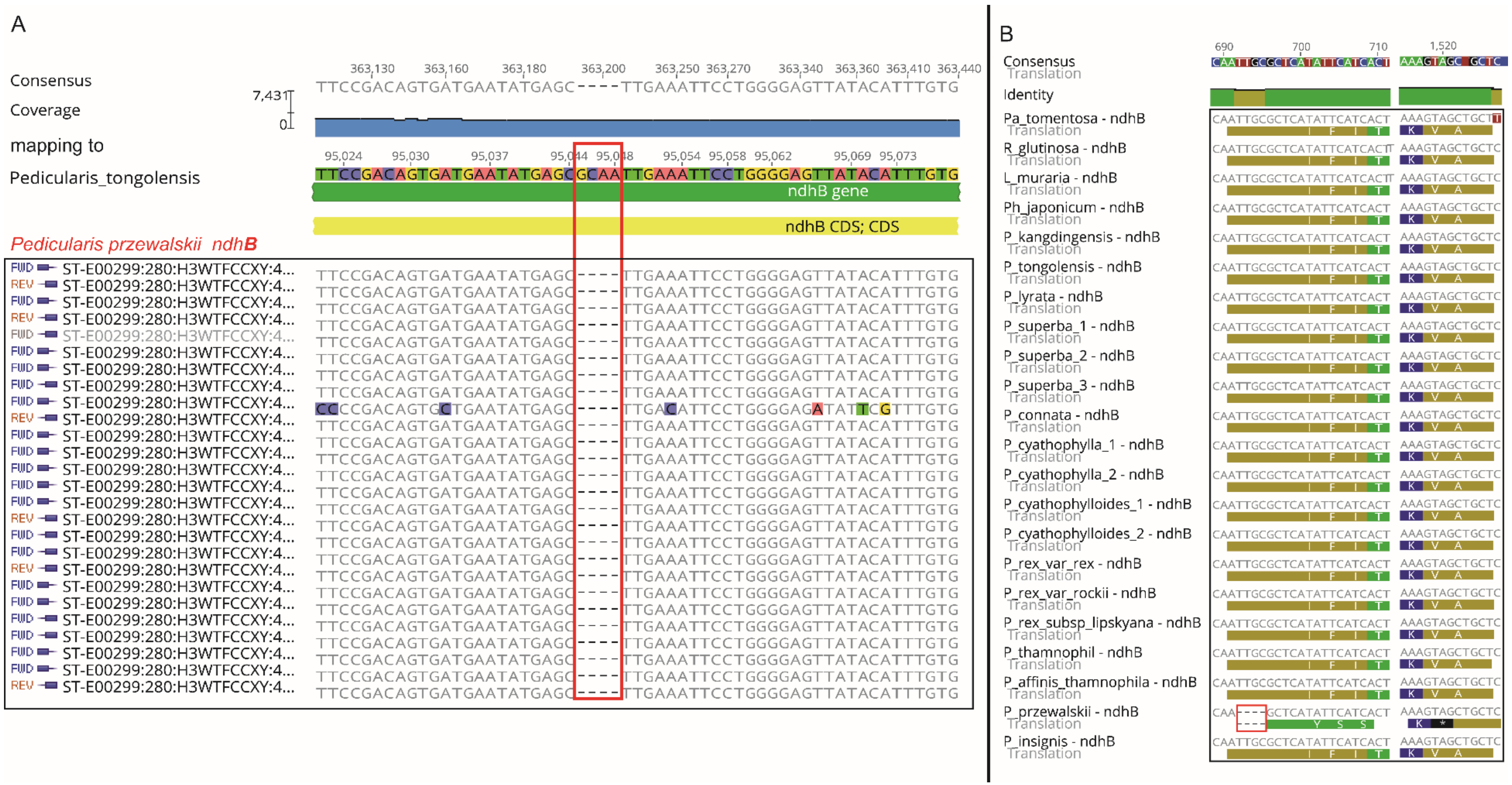

Figure S9. Raw reads of *Pedicularis przewalskii* mapping to the plastome of *Pedicularis tongolensis* as the reference in the *ndh*B gene region showed that the raw reads of *P. przewalskii* lost four bases (GCAA) in the *ndh*B gene (A). The *ndh*B gene of *P. przewalskii* lost four bases (GCAA) (red box) in comparison with other species in the *ndh*B gene alignment(B).

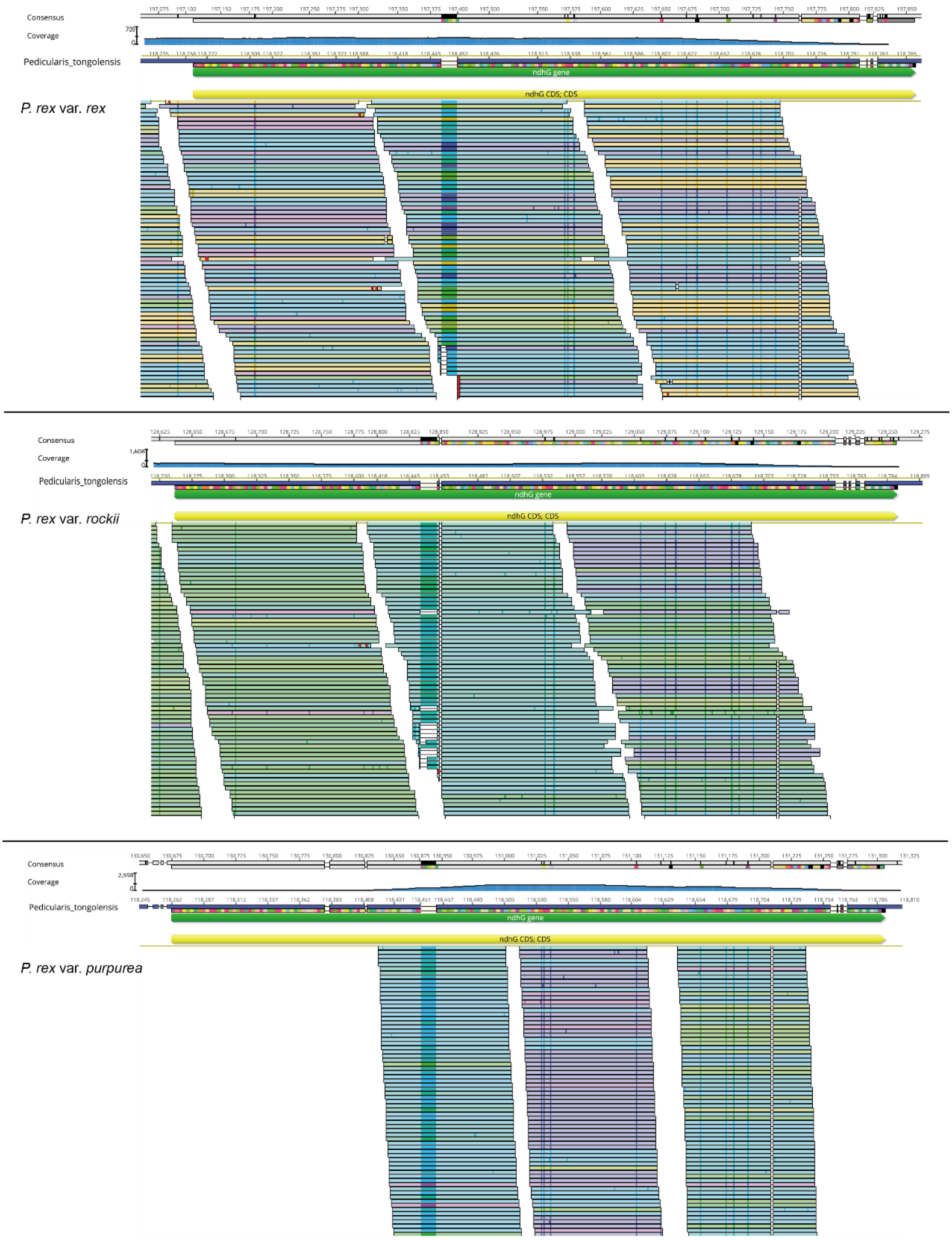

Figure S10. Raw reads of three varieties of *Pedicularis rex* mapping to the plastome of *Pedicularis tongolensis* as the reference in the *ndh*G gene region showing that the *ndh*G gene of *Pedicularis rex* var. *purpurea* was fragmented.

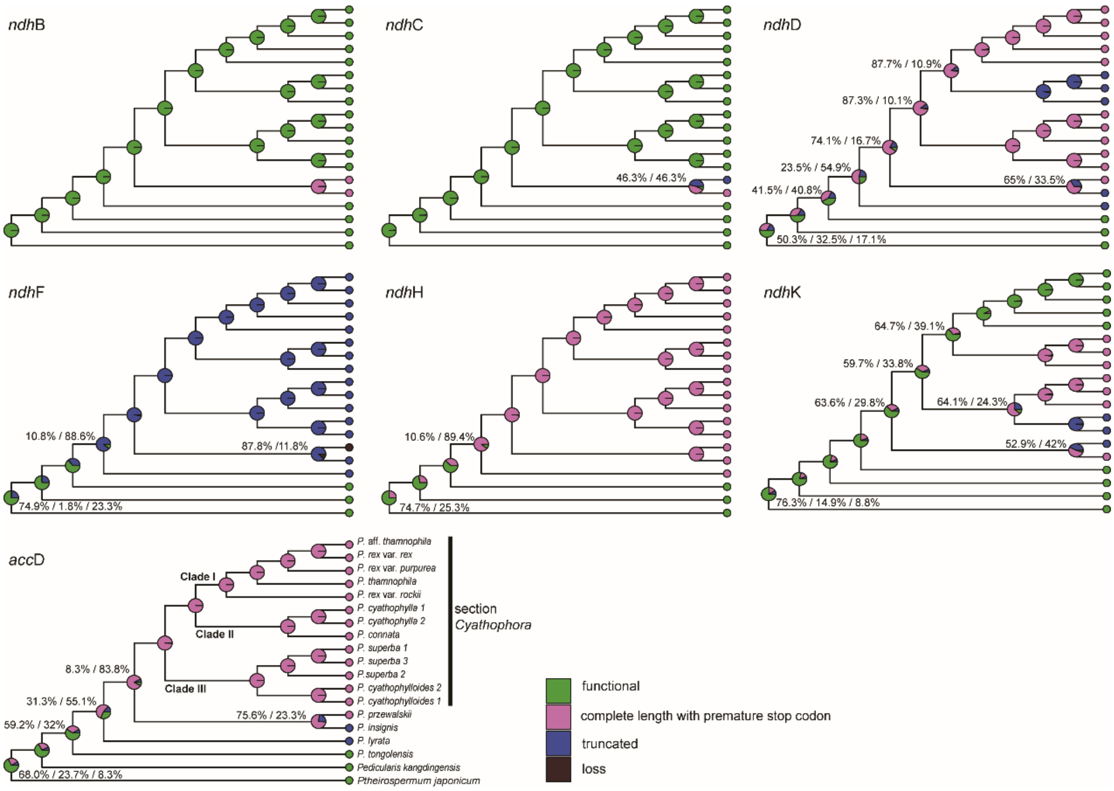

Figure S11. Ancestral state reconstruction of *acc*D, NDH (B/C/D/F/H/K) genes in *Pedicularis* using the “ER” likelihood model.

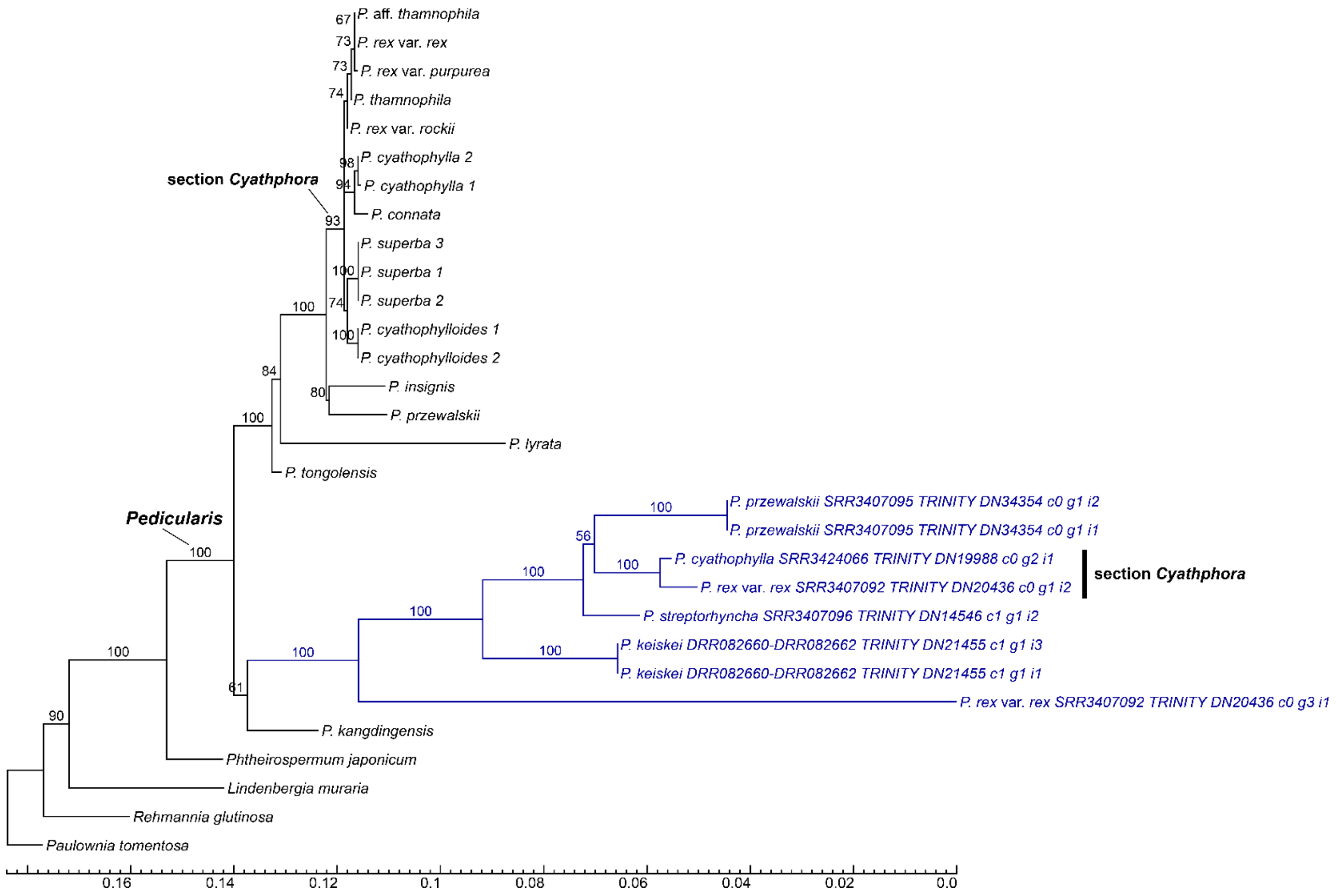

Figure S12. Phylogenetic relationship between plastid and nuclear *acc*D genes in *Pedicularis*. ML Bootstrap values are presented above branches. The bottom scale bar represents the number of substitutions per site.

Table S1. Summary of published plastome features of hemiparasitic plants.

Order	Genus	Species	NCBI no.	Plastome size (bp)	GC (%)	NDH genes		Other genes		References
						Pesudogene	Loss	Pesudogene	Loss	
Lamiales	*Aureolaria*	*virginica*	MF780870	153,547	38.4					Frailey et al. 2018
	Buchnera	*americana*	MF780871	166,596	37.8	A, B, C, D, E, G, I, J, K	F, H			Frailey et al. 2018
	Castilleja	*paramensis*	KT959111	152,926	38.2	A, D, F, H, G, J	I			Fan et al. 2016
	Pedicularis	*alaschanica*	MK795426	146,989	38.4	B, D, E, H, G, J, K, C,I	A,F			Wu et al. 2019
		ishidoyana	NC_029700	152,517	38.1	B, C, G, I, J, K	A, H, D, F	*rpo*A, *ccs*A		Cho et al. 2018
	Schwalbea	*americana*	HG738866	160,911	38.1	A, D, F, G		*acc*D		Wicke et al. 2013
	Striga	*aspera*	MF780872	185,932	38.0	A, B, D, E, F, I, J, K	C, G, H	*ycf1*, *ycf2*		Frailey et al. 2018
		forbesii	MF780873	190,233	37.8	A, B, C, D, E, F, G, H, I, J, K				Frailey et al. 2018
		hermonthica	MF780874	186,418	38.0	A, B, D, E, F, I, J, K	C, G, H	*ycf1*, *ycf2*		Frailey et al. 2018
	Triphysaria	*versicolor*	KU212369	152,448	38.2		B, D	*rpo*B, *C1*, *C2*, *pet*B		Wicke et al. 2016
Laurales	*Cassytha*	*filiformis*	MF939337	114,623	36.9	B, D, E, F, H	A, C, G, I, J, K			Song et al. 2017

		capillaris	MF939338	114,963	36.9	B, D, E, F, H	A, C, G, I, J, K			Song et al. 2017
**Santalales**	*Dendrotrophe*	*varians*	NC_039391	140,666	37.8	B	A, C, D, E, F, G, H, I, J, K	*inf*A		Shin and Lee. 2018
	Dendrophthoe	*pentandra*	MN175255	115,721	37.0		A, B, C, D, E, F, G, H, I, J, K		*rps15, rps16, rpl32, rpl36, inf*A; *trnG-UCC, H-GUG,I-GAU, K-UUU, V-UAC*	Guo and Ruan. 2019
	Helixanthera	*parasitica*	NC_039375	124,881	36.5	B	A, C, D, E, F, G, H, I, J, K	*rpl16*	*infA, rpl32, rps15, 16; trnA-UGC, I-GAU, K-UUU,V-UAC,G-UCC*	Shin and Lee. 2019
	Erythropalum	*scandens*	NC_036759	156,154	38.0					Chen et al. 2019
	Loranthus	*tanakae*	MN414178	123,397	36.9	B	A, C, D, E, F, G, H, I, J, K			Chen et al. 2019
	Macrosolen	*cochinchinensis*	NC_039376	122,986	36.6	B	A, C, D, E, F, G, H, I, J, K	*inf*A	*rpl32, 36; rps15, 16; trnA-UGC, I-GAU, K-UUU,V-UAC,G-UCC*	Shin and Lee. 2019
	Osyris	*alba*	KT070882	147,253	37.7	B, C, D, E, K, A, G, H	F, I, J	*inf*A		Petersen et al. 2015
	Schoepfia	*jasminodora*	NC_034228	118,734	38.1	A	B, C, D, E, F, G, H, I, J, K	*ycf15, trnL-CAA*	*trnV-UAG, trnG-UCC*	Su and Hu. 2016
	Taxillus	*chinensis*	KY996492	121,363	37.3		A, B, C, D, E, F, G, H, I, J, K	*ycf15, rpl2, rpl16*	*inf*A, *rpl32, rps15, 16; trnA-UGG, G-UCC, H-GUG, L-GAU, K-UUU, L-UAA, V-UAC*	Li et al. 2017
		vestitus	MN175257	122,200	37.3		A, B, C, D, E, F, G, H, I, J, K		*rps15, rps16, rpl32, inf*A; *trnG-UCC, H-GUG,K-UUU, V-UAC*	Guo et al. 2019
		sutchuenensis	KY996493	122,562	37.3		A, B, C, D, E, F, G, H, I, J, K	*ycf15, rpl2, rpl16*	*inf*A *rpl32, rps15, 16;trnA-UGG, G-UCC, H-GUG, L-GAU, K-UUU, L-UAA, V-UAC*	Li et al. 2017
	Tolypanthus	*maclurei*	MH922027	123,581	36.8	B	A, C, D, E, F, G, H, I, J, K			Chen et al. 2019

	Elytranthe	*Albida*	MN175256	128,658	37.6		A, B, C, D, E, F, G, H, I, J, K		*rps15, rps16, rpl32, inf*A; *trnG-UCC, trnV-UAC*	Guo et al. 2019
	Viscum	*album*	KT003925	128,921	36.4	B	A, C, D, E, F, G, H, I, J, K	*ccs*A, *mat*K	*inf*A, *rpl33*; *trnV-UAC, trnG-UCC*	Petersen et al. 2015
		crassulae	KT070881	126,064	36.4	B	A, C, D, E, F, G, H, I, J, K		*inf*A, *rpl33*; *trnV-UAC, trnG-UCC*	Petersen et al. 2015
		minimum	KJ512176	131,016	36.2	B	A, C, D, E, F, G, H, I, J, K		*inf*A, *rpl33, trnV-UAC*	Petersen et al. 2015
		rotundifolia	NC_023790	160,891	37.4					Chen et al. 2019

References

Chen X, Fang D, Wu C, et al (2019) Comparative plastome analysis of root- and stem-feeding parasites of Santalales untangle the footprints of feeding mode and lifestyle transitions. *Genome Biology and Evolution*. 12: 3663-3676.

Cho WB, Choi BH, Kim JH, et al (2018) Complete plastome sequencing reveals an extremely diminished SSC region in hemiparasitic *Pedicularis ishidoyana* (Orobanchaceae). *Annales Botanici Fennici*. 55: 171-183.

Fan W, Zhu A, Kozaczek M, et al. (2016) Limited mitogenomic degradation in response to a parasitic lifestyle in Orobanchaceae. Scientific reports, 3:36285

Frailey DC, Chaluvadi SR, Vaughn JN, et al (2018) Gene loss and genome rearrangement in the plastids of five hemiparasites in the family Orobanchaceae. *BMC Plant Biology.* 18: 30.

Guo XR, Ruan ZJ (2019) Characterization of the complete plastome of *Dendrophthoe pentandra* (Loranthaceae), a stem hemiparasite, *Mitochondrial DNA Part B*. 4: 3099-3100

Guo XR, Ruan ZJ, Guangfei Zhang GF (2019) The complete plastome of *Taxillus vestitus* (Loranthaceae), a hemiparasitic plant, *Mitochondrial DNA Part B*. 4: 3188-3189.

Guo XR, Ruan ZJ (2019) The complete chloroplast genome of *Elytranthe albida* (Loranthaceae), a hemiparasitic shrub. *Mitochondrial DNA Part B*. 4: 3112-3113.

Li Y, Zhou JG, Chen XL, et al (2017) Gene losses and partial deletion of small single-copy regions of the chloroplast genomes of two hemiparasitic *Taxillus* species. *Scientific Reports*. 7: 12834.

Petersen G, Cuenca A, Seberg O (2015) Plastome evolution in hemiparasitic mistletoes. *Genome Biology and Evolution*. 7: 2520-32.

Song Y, Yu WB, Tan Y, et al (2017) Evolutionary comparisons of the chloroplast genome in Lauraceae and insights into loss events in the Magnoliids. *Genome Biology and Evolution*. 9: 2354-2364.

Shin HW, Lee NS (2018) Understanding plastome evolution in hemiparasitic Santalales: Complete chloroplast genomes of three species, *Dendrotrophe varians*, *Helixanthera parasitica*, and *Macrosolen cochinchinensis*. *PLoS One*. 13: e0200293.

Su HJ, Hu JM (2016) The complete chloroplast genome of hemiparasitic flowering plant *Schoepfia jasminodor*. *Mitochondrial DNA Part B*. 1: 767-769.

Wicke S, Muller KF, dePamphilis CW, et al (2016) Mechanistic model of evolutionary rate variation en route to a nonphotosynthetic lifestyle in plants. *Proceedings of the National Academy of Sciences of the United States of America*. 113: 9045-9050.

Wicke S, Muller KF, de Pamphilis CW, et al (2013) Mechanisms of functional and physical genome reduction in photosynthetic and nonphotosynthetic parasitic plants of the broomrape family. *Plant Cell*. 25: 3711-3725.

Wu CY, Fang DM, Wei JP, et al. (2019) The complete chloroplast genome of *Pedicularis alaschanica* (Orobanchaceae). *Mitochondrial DNA Part B*. 4: 2197-2198.

Table S2. The molecular information and mapping results of all plastid genomes.

DNA no.	Collection no.	Species	Total reads	plastid reads	plastid reads ratio (%)	aligned
Pedicularis* sect. *Cyathophora						
Series *Cyathophyllae*						
P109	Yu et al. HW10208	*P. cyathophylla 1*	22,546,064	1,234,581	5.48%	1195.27±151.55
P578	Yu et al. LIDZ1195	*P. cyathophylla 2*	25,281,062	2,937,246	11.62%	2829.38±329.88
Series *Cyathophylloides*						
P110	Yu et al. YWB132	*P. cyathophylloides 1*	19,570,002	245,290	1.25%	238.14±35.38
P747	Boufford et al. 41478	*P. cyathophylloides 2*	13,840,860	295,531	2.14%	286.66±43.7
Series *Reges*						
P787	Jiang et al. AZY20	*P. rex* var. *rex*	22,443,994	327,235	1.46%	318.64±74.62
S12453	Zhang et al. 11CS3092	*P. rex* var. *rockii*	16,726,564	623,162	3.73%	568.13±266.13
P67	Yu et al. YWB201507163	*P. rex* var. *purpurea* (≡*P. rex* ssp. *lipskyana*)	45,232,898	1,564,115	3.46%	1018.76±215.04
P68	Yu et al. YWB201507262	*P.* affinis *thamnophila*	46,550,092	2,450,178	5.26%	1596±233.27
P113	Yu et al. HW10234	*P. thamnophila*	16,418,052	793,379	4.83%	807.79±151.44
Series *Superbae*						
P772	Rock 16742	*P. connata*	23,123,144	379,890	1.64%	360.84±69.51
S12548	Cai et al. 11CS3251	*P. superba 1*	15,907,124	1,491,578	9.38%	1358.19±520
S12628	Zhang et al. 11CS3397	*P. superba 2*	21,620,436	671,983	3.11%	612.13±255.01
S12737	Zhang et al. 11CS3607	*P. superba 3*	14,292,578	233,681	1.63%	212.87±75.31
**Other *Pedicularis***						
P112	Yu et al. LIDZ1235	*P. przewalskii*	12,139,564	2,885,321	23.77%	2947.41±978.31
P111	Gao et al., GLM123699	*P. insignis*	35,653,814	3,938,702	11.05%	3996.52±1182.74
P069	Yu et al. YWB201507258	*P. lyrata*	35,025,234	882,242	2.52%	594.45±110.48
P066	Yu et al. YWB201507225	*P. tongolensis*	11,243,246	1,567,846	13.94%	1032.41±160.94
P062	Yu et al. YWB201507208	*P. kangdingensis*	42,947,980	2,086,941	4.86%	1376.68±284.74
**Non-*Peddicularis***						
DRR082673	SRA data	*Phtheirospermum japonicum*	156,452,544	12,989,849	8.30%	7622±936.66
S00403	Meng et al. MJ563	*Lindenbergia muraria*	19,004,876	592,280	3.12%	576.14±95.90
J2387	Bian et al. BianFH0288	*Rehmannia glutinosa*	22,410,154	2,706,599	12.08%	2638.3±278.36
SRR6940033	SRA data	*Paulownia tomentosa*	58,914,346	2,622,342	4.45%	2539.39±336.28

Table S3. Wilcoxon test of plastome length, GC content, the number of unique CDS genes and pseudogenes among three groups.

	Other *Pedicularis*		non-*Pedicularis*		Characters
	W	p-value	W	p-value	
P.* section *Cyathophora	65.0	0.002**	34.0	0.396	**Complete size**
**Other *Pedicularis***			20.0	0.016*	
P.* section *Cyathophora	50.0	0.093	0.0	0.004**	**LSC size**
**Other *Pedicularis***			20.0	0.016*	
P.* section *Cyathophora	65.0	0.002**	52.0	0.004**	**IR size**
**Other *Pedicularis***			14.0	0.413	
P.* section *Cyathophora	0.0	0.002**	0.0	0.004**	**SSC size**
**Other *Pedicularis***			20.0	0.016*	
P.* section *Cyathophora	10.5	0.019*	44.0	0.031*	**Complete GC%**
**Other *Pedicularis***			1.0	0.034*	
P.* section *Cyathophora	39.0	0.362	52.0	0.000**	**LSC GC%**
**Other *Pedicularis***			1.0	0.033*	
P.* section *Cyathophora	0.0	0.001**	0.0	0.003**	**IR GC%**
**Other *Pedicularis***			6.0	0.385	
P.* section *Cyathophora	65.0	0.002**	52.0	0.004**	**SSC GC%**
**Other *Pedicularis***			3.5	0.14	
P.* section *Cyathophora	21.0	0.261	0.0	0.003**	**Unique CDS genes**
**Other *Pedicularis***			20.0	0.015*	
P.* section *Cyathophora	34.0	0.92	52.0	0.003**	**Pseudogenes**
**Other *Pedicularis***			0.0	0.015*	

**, $P < 0.01$; *, $P < 0.05$

Table S4. Reconstructing the ancestral state of the 13 pseudogenes using R package.

Gene	0	1	2	3	Log-likelihood
accD	67.96%	23.71%	8.33%		-11.38922
ccsA	15.41%	84.59%			-9.044556
ndhA	65.82%	7.77%	26.42%		-11.40429
ndhB	99.81%	0.19%			-3.64762
ndhC	99.57%	0.21%	0.21%		-8.276355
ndhD	50.35%	32.53%	17.12%		-13.43685
ndhE	44.20%	43.23%		12.57%	-15.02597
ndhF	74.86%		23.30%	1.84%	-10.30267
ndhG	43.29%	23.54%	15.03%	18.14%	-20.81489
ndhH	74.68%	25.32%			-4.99264
ndhI	44.27%	31.75%		23.98%	-13.59232
ndhJ	82.05%	17.95%			-8.236362
ndhK	76.29%	14.95%	8.77%		-15.3453

Four states for each gene: 0, functional; 1, full sequence with premature stop codon; 2, truncated gene; 3, complete loss.
